# Voglibose Attenuates Amyloid Beta–Induced Memory Deficits in a Rodent Model: A Potential Alzheimer’s Therapy via Wnt Signaling Modulation

**DOI:** 10.1007/s12035-025-05047-5

**Published:** 2025-05-17

**Authors:** Suman Manandhar, Prasada Chowdari Gurram, Anusha Govindula, Shama Prasada Kabekkodu, K Sreedhara Ranganath Pai

**Affiliations:** 1 Department of Pharmacology, Manipal College of Pharmaceutical Sciences, Manipal Academy of Higher Education, Manipal, Karnataka 576104 India; 2 Department of Cell and Molecular Biology, Manipal School of Life Sciences, Manipal Academy of Higher Education, Manipal, Karnataka 576104 India

**Keywords:** Wnt signaling pathway, Aβ_25-35_, Behavioral study, Diabetes, Alzheimer’s disease, Drug repurposing

## Abstract

Disruption of the Wnt signaling pathway (WSP), a highly conserved pathway essential for growth and organ development, has been proven to play a role in the pathogenesis of Alzheimer’s disease (AD). This study focused on repurposing the FDA-approved drug, Voglibose to target the DKK1-LRP6 site with the goal of upregulating WSP in in vitro as well as rodent model of AD. Based on our previous computational approach, Voglibose was evaluated for the DKK1 binding, neuroprotective effects were examined using SHSY5Y cells, while WSP activation was analyzed through RTPCR in the HEK293/LRP6 cell line. Rodent model of AD was developed using intracerebroventricular administration of Aβ_25-35_. Male Wistar rats were randomly assigned to receive oral doses of Voglibose (1 and 10 mg/kg) for 28 days, after which behavioral assessments, biochemical analyses, RT-PCR, and histopathological evaluations were conducted. Voglibose showed significant reduction in the DKK1 binding, neuroprotective property in SHSY5Y as well as activation of WSP in LRP6 overexpressed HEK293 cells. There was a significant decrease in the island latency in rats treated with lower dose (*p* < 0.01) and higher dose (*p* < 0.05) of Voglibose when compared to the disease control rats. Similarly, in the behavioral tests, Voglibose significantly improved cognition. The deposition of amyloid plaques was found to be considerably more in the disease control rats which got reduced in the treatment groups as observed in the histopathological slides stained with Congo red. Significant alterations in mRNA levels and protein expression of glycogen synthase kinase-β (GSK-3β), β-catenin (β-cat) was observed in rat brain homogenates indicating upregulation of WSP. In conclusion, Voglibose demonstrated significant neuroprotective potential in a cell line study and showed potential cognitive benefits in a rat model of AD. Furthermore, its ability to activate WSP highlights its immense potential as AD therapeutic to enhance memory and modulate key neuroprotective mechanisms.

**Supplementary Information:**

The online version contains supplementary material available at 10.1007/s12035-025-05047-5.

## Introduction

Alzheimer’s disease (AD) is a progressive neurological disorder characterized by marked cognitive decline with poor prognosis [1]. As the global population ages, the prevalence of this devastating condition is on the rise, with estimates suggesting it will impact 131.5 million cases by 2050. This underscores the need to explore its complexities and seek innovative research and intervention strategies [2, 3]. The primary pathological features of AD include intracellular neurofibrillary tangles and extracellular senile plaques, particularly those composed of aggregated amyloid-β and hyper-phosphorylated tau [4]. Although the exact molecular mechanisms driving neurodegeneration in AD remain unclear, studies suggest that oxidative stress and neuronal apoptosis are vital factors in the disease’s development [5, 6]. AD has been linked to the amyloid hypothesis, neuroinflammation, oxidative stress, insulin resistance, and aberrant mitochondrial dysfunction [7]. Furthermore, the onset of AD has been connected to the loss of the typical Wnt signaling pathway (WSP), an evolutionary conserved signaling involved in growth and organ development [8–10]. Increased expression of the Wnt antagonist dickkopf-related protein 1 (DKK1), malfunctioning of the Wnt coreceptor low-density lipoprotein receptor–related protein 6 (LRP6), and activation of glycogen synthase kinase-3β (GSK-3β) have all been shown in AD brains. Research carried both in vitro and in vivo has revealed that inhibiting WSP causes amyloidogenic breakdown of the precursor protein amyloid (APP), which elevates the production and build-up of Aβ_1–42_ [11].

AD and cognitive dysfunction are more common in elderly diabetic people, it has been proposed that AD, also known as “brain-specific diabetes” or “Type 3 diabetes”, is brought on by insulin resistance [12]. There has been speculation that insulin resistance and compromised insulin signaling may play a role in the possible relationship between type 2 diabetes and AD in the central nervous system. Insulin resistance and persistent inflammation are the hallmarks of diabetes type II, a chronic illness that typically manifests in old life [13]. Genetic studies have revealed that the WSP is associated with the chromosomal regions which are most vulnerable to the onset of diabetes [14, 15]. This pathway is essential for the growth, operation, and secretion of insulin by the pancreatic islet cells [16]. Individuals with a missense mutation in the *LRP6* gene typically exhibit impaired insulin signaling in skeletal muscle, resulting in hyperinsulinemia, and poor insulin sensitivity [17]. Insulin-resistant β-cells in diabetes cause WSP to be downregulated, which reduces insulin production and causes pancreatic islet cell failure. Data from Centre for Disease Control and Prevention’s wide-ranging online data for epidemiological research (CDC-WONDER) in the USA revealed a significant rise in type 2 diabetes mellitus and AD comorbidity-related mortality among adults aged 65, particularly Hispanics, women, and rural residents. These findings highlight the need for targeted interventions in vulnerable populations [18]. Furthermore, the incidence densities of AD among diabetic men and women were 0.82 and 1.15 per 1000 person-years, respectively with a significantly higher hazard ratio across all ages and both sexes. Notably, older diabetic women exhibited the highest hazard ratio for AD [19]. Also, recent research highlights that insulin resistance contributes to AD pathogenesis through impaired insulin signaling, inflammation, and disrupted glucose metabolism. This link underscores the heightened AD risk in diabetic patients and suggests potential treatment strategies including metabolic interventions and repurposing diabetic medications for AD [20]. As the main pathogenic mechanism tying AD and diabetes mellitus together, numerous research have concentrated on insulin, its signaling, and resistance in both peripheral and central tissues [21]. GSK-3β activity is inhibited when insulin attaches to its receptor and initiates insulin signaling, which in turn stimulates the phosphoinositide 3-kinases (PI3 K) and AKT signaling pathway. GSK-3β is overexpressed in AD and plays a role in β-catenin degradation as well as WSP inactivation [22–24].

The use of antidiabetic drugs as a possible treatment for AD has been the subject of numerous investigations, both in preclinical and clinical settings. Drugs that reduce postprandial glucose levels with a decreased risk of hypoglycemia may be able to stop the cognitive loss that occurs in elderly diabetics. Different classes of anti-diabetic medications, including intranasal insulin, glucagon-like peptide receptor agonists, biguanides, thiazolidinediones, and dipeptidyl peptidase-4 inhibitors, have been shown to improve memory and reduce neuroinflammation and oxidative stress in AD patients [25, 26]. Voglibose, a α-glucosidase inhibitor commonly used to manage postprandial hyperglycemia, acts by inhibiting α-glucosidase (maltase) enzyme leading to the breakdown of carbohydrates. Voglibose is commonly used to treat type 2 diabetic patients, and its activity mediated through inhibition of insulin receptors mediated through mitochondrial dysfunction involving alterations in electron transfer chain complex activities, resulting in oxidative stress and neuronal apoptosis. Inflammatory responses and their mediators play a significant role in the progression of neurodegenerative diseases, emphasizing the need to explore neuroprotective agents that may help delay disease advancement. Voglibose has been investigated for its effects on oxidative stress and various related conditions [27, 28]. Voglibose has been repurposed for its neuroprotective activity through attenuation of polyol pathway [29], attenuation of cognitive impairment mediated through neuroinflammation and mitochondrial dysfunction in streptozocin-induced AD model. [28]

The novelty of the current study was to evaluate the neuroprotective activity of Voglibose using in vitro as well as in vivo rodent model of AD through modulation of WSP. In our previous study, we have shown the strong binding of Voglibose to LRP6 protein using computational tools like docking and molecular dynamic simulation [30]. Using computational tools, we had identified Voglibose as having a strong docking score and binding affinity, to LRP6 demonstrating its potential to activate WSP and potentially improve cognition. In the present study, we have shown the neuroprotective activity of Voglibose in SHSY5Y cells and activation of canonical WSP using LRP6 overexpressed HEK293 cells after Voglibose treatment. Additionally, we have shown Voglibose prevented the binding of DKK1 to LRP6 by using DKK1 binding assay which led to activation of WSP. Further, we have evaluated the neuroprotective potential of Voglibose in ICV Aβ_25–35_-induced AD rat model using behavioral parameters to show cognitive restoration, histopathological alteration in brain amyloid level and markers related to WSP.

## Methodology

### Cell Culture

SHSY5Y, HEK-293, and HEK-293 overexpressing LRP6 (HEK293/LRP6) were cultured in Dulbecco’s modified eagle’s medium (DMEM) (Sigma Aldrich, India) supplemented with 10% fetal bovine serum (FBS) (Invitrogen), 1% penicillin/streptomycin (Sigma Aldrich, India), and 50 µg/ml of hygromycin for HEK293/LRP6 in a 5% CO_2_ at 37 °C.

### Differentiation of SHSY5Y Neuroblastoma to Neuronal Culture

SHSY5Y cells were maintained in a DMEM medium containing 10% FBS and 1% penicillin streptomycin antibiotic at 37 °C. For differentiation 10,000 cells were seeded in 96-well plate and incubated at 37 °C for 24 h. After 1 day, cells were treated with 10 µM concentration of retinoic acid (RA) [31, 32]. Every alternate day the media was replaced with the fresh media containing 10 µM RA for 5 days [33, 34]. The extent of differentiation was evaluated based on the growth of the neurites.

### Evaluation of Neuroprotective Activity in Differentiated and Undifferentiated SHSY5Y Cells

SHSY5Y cells (10,000) were seeded in 96-well plate and were differentiated with RA for 5 days. Both the differentiated and undifferentiated SHSY5Y cells were treated with 80 mM of glutamate [35] and different concentration (100, 50, 25, 12.5, 6.25, 3.12, 1.56 µM) of Voglibose then incubated for 48 h. After 48-h duration, the media was removed, and cells were treated with 0.5 mg/ml of 3-(4,5-dimethylthiazol-2-yl)−2,5-diphenyltetrazolium bromide (MTT) for 4 h at 37 °C [36]. The formazan crystals once formed were dissolved in dimethyl sulfoxide (DMSO) followed by the measurement of absorbance at 570 nm in microplate reader (Biotek Epoch2 Plate reader). MTT assay was done to identify the dose at which optimum neuroprotection was observed [37].

### Overexpression of LRP6 Protein in HEK293 Cells

The full-length clone DNA of human LRP6 with pCMV3-C-flag vector (Cat: HG 17052-CF; Sino biologicals Inc.) was obtained from Sino biologicals Inc., China. To create plasmid stocks, *E. coli* cells that were capable of being transformed were utilized. The given cells underwent a transformation process using 0.2 µg of plasmid as per the instructions provided by the supplier. Subsequently, one colony of the transformed cells was used to inoculate 150 mL of lysogeny broth (LB) medium. The culture was then allowed to grow for 16 h at 37 °C with continuous shaking at a rate of 8 × *g*. The medium used for the process contained kanamycin at a final concentration of 50 µg/ml [38]. The isolation of plasmid was done from *E. coli* using Maxiprep/Mini prep method. HEK293 cells were grown at 70–80% confluence and transfected with 1 µg of the pCMV3 c Flag LRP6 or pCMV3 empty vector in Opti-MEM media using lipofectamine 2000 reagent (Cat: 11,668,027; Invitrogen, USA) followed by incubation for 48 h at 37 °C [39]. The transfected cells were analyzed by using western blot technique to confirm the overexpression of LRP6 protein.

### Evaluation of Cytotoxicity in HEK293/LRP6 Cells Using MTT Assay

HEK293/LRP6 cells (5000) were seeded in 96-well and were treated with the different concentration of the Voglibose (100, 50,25, 12.5, 6.25, 3.12, 1.56 µM) and incubated for 48 h followed by MTT assay to identify the dose at which optimum toxicity was observed. After 48 h duration, the cells were treated with 0.5 mg/ml of MTT for 4 h at 37 °C [40]. Formazan crystals which were formed formazan were dissolved in DMSO followed by the measurement of absorbance at 570 nm in microplate reader (Biotek Epoch2 Plate reader).

### DKK1 Binding Assay

DKK1 binding assay was carried out in accordance with the methodology previously described by Priestley et al. [38]. The experiment involved seeding HEK293/LRP6 cells onto poly-d-lysine-coated coverslips 24 h before the experiment. The cells were then incubated with 0.5 µg/ml of lyophilized DKK1 protein, in the presence of WAY262611 (5 μM), or Voglibose (15 μM and 25 μM) for a period of 48 h at 37 °C. Following the incubation, the cells were washed and then fixed using 3% paraformaldehyde for 15 min at room temperature. The cells were subsequently blocked and permeabilized with triton × 100 and phosphate buffered saline (PBS)/0.1% bovine serum albumin (BSA) and then incubated with the primary rabbit anti-DKK1 antibody, and fluorescent secondary antibody Alexa488, which was diluted in blocking solution. Finally, the cell nuclei were counterstained with 4,6 diamidino-2-phenylindole (DAPI), and the images were collected using a fluorescence microscope, and the florescence intensity was measured using ImageJ software.

### qPCR Analysis for Analysis of Gene Expression Related to WSP

HEK293/LRP6 cells were seeded in 12-well plate at density of 1.5 × 10^5^ then incubated for 48 h with various concentrations of specific drugs. Afterwards, the RNAiso plus reagent (Cat: 9108; Takara Bio Inc.) was used to isolate total RNA from the cell culture, following the instructions provided by the manufacturer [41] (Takara Bio Inc.). Reverse transcription was done using 1 µg of total RNA with a primescript reverse transcription kit (Cat: RR037 A; Takara Bio Inc.). Primers used in this study are human *GSK3B* (sense, 5’-GGAACTCCAACAAGGGAGCA-3’, antisense, 5’-TTCGGGGTCGGAAGACCTTA-3’), *CTNNB1* for qPCR (sense, 5′-CCTCCAGGTGACAGCAATCAG-3′, antisense, 5′-GCCCTCTCAGCAACTCTACAG-3′) *MYC* for qPCR (sense, 5′- TACCCTCTCAACGACAGCAG-3′, antisense, 5′- TCTTGACAT TCTCCTCGGTG-3′), *GAPDH* (sense, 5′-CAGTCAGCCGCATCTTCTTTT-3′, antisense, 5′-GTGACCAGGCGCCCAATAC-3′). *GAPDH* was used as housekeeping gene control. For the PCR, 10 µl of cDNA was utilized, and three replicate reactions were conducted for every sample utilizing TB Green TM Premix Ex (Cat: RR820 A; Takara Bio Inc.) in conjunction with gene-specific primers on a CFX96 instrument (Biorad). The amount of RNA present was normalized to *GAPDH* content, and the expression of genes was determined using the 2 ^− ΔΔCt method.

### Animals

The present study utilized male Wistar Rats weighing between 250 and 300 g, which were obtained from the inbred strains of the central animal research facility at Manipal academy of higher education. Ethical clearance was obtained from the institutional ethics committee (IAEC/KMC/91/2021). The rats were housed in propylene cages with three rats per cage and provided with unrestricted access to food and water while being kept at an optimal temperature with a relative humidity of 55% and a 12-h light/dark cycle. All animal care and handling were performed in accordance with the institutional ethics committee’s guidelines.

### Amyloid Beta 25–35 (Aβ_25–35_) Oligomer Preparation

The Aβ_25–35_ peptide used in this study was obtained from Apexbio Technology LLC (Cat: A1039). Aβ_25–35_ oligomers were generated using the method described by Stine et al. [42]. To resuspend the lyophilized peptide, the first step involved treating it with 1,1,1,3,3,3-hexafluoro-2-propanol (HFIP) in a chemical fume hood. The peptide was diluted in 100% HFIP to 1 mM. The HFIP was allowed to evaporate in the fume hood, and the resulting clear peptide films were vacuum-dried and stored at − 20 °C. Just before use, the HFIP-treated aliquots were carefully resuspended in anhydrous DMSO to 5 mM using pipette mixing and bath sonication for 10 min. To prepare the Aβ_25–35_ oligomers, a solution of 5 mM Aβ_25–35_ in DMSO was diluted in ice-cold PBS to a concentration of 100 μM. The solution was then vortexed for 30 s and left to incubate at 4 °C for 3 days.

### Atomic Force Microscopy Analysis

After 3 days of incubation at 4 °C, the aliquot of Aβ was diluted to a concentration of 1000 nM with PBS, and 10 µl of the diluted Aβ was dispersed on freshly cleaned mica disc. Following a process of drying inside an antistatic container at a temperature of 4 °C in a dry atmosphere, an atomic force microscopy (AFM) Innova system (Bruker, Model No:1B342, Billerica, MA, USA) was used to conduct observations in the air, under room temperature conditions and with ambient humidity [43]. The AFM method employed the tapping mode to examine mica discs.

### Intracerebroventricular Aβ_25–35_ Treatment in Rats

**Fig. 1:**
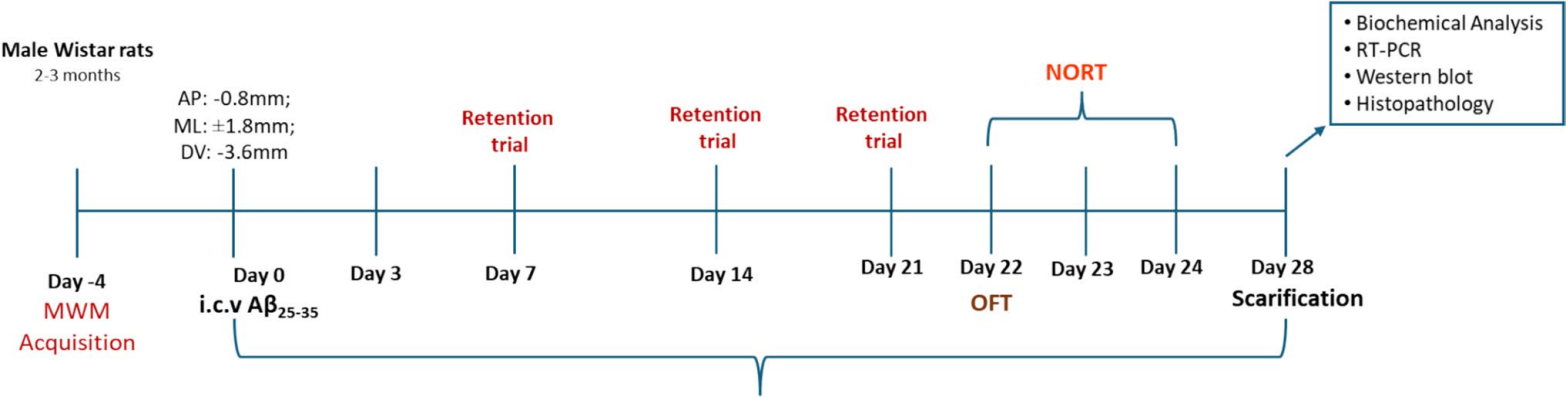
Representation of the schedule and treatment plan of animal model of AD administered with amyloid dose intracerebroventricularly followed by treatment with Voglibose

Stereotaxic surgery was performed as per the method described by Satrker S et al. [44], Kumar, Dogra, and Prakash [45]. Amyloid beta (25–35) administration was done at different doses using the Stereotaxis apparatus, with stereo drive software preloaded with rat brain atlas. Animals were administered ketamine (80 mg/kg) and xylazine (10 mg/kg) for maintenance of anesthesia. The animal’s head was aligned in the frame of the apparatus, and an incision was made along the midline of the scalp. The point on the skull where the syringe points to is marked, and a hole was drilled in the skull for ICV administration. Five microliters of PBS or Aβ was injected into the lateral ventricle of the brain in the co-ordinates (AP, − 0.8 mm; ML, 1.8 mm; DV, − 3.6 mm) using Hamilton microsyringe and was placed for 2 min at the injection place to avoid backflow. The scalp was closed with suture, and the antibacterial cream was applied to the incision to prevent any infection. The treatment schedule and groupings of the rats used for the standardization of rodent model of ICV Aβ_25–35_ has been represented in Table 1 and Fig. 1. After the surgery, animals were then housed in cages with soft bedding along with provision for food and water inside the cage.

**Table 1:** Grouping of the animals with the treatment and dosing plan

S.no	Groups (*n* = 6)	Dose
1)	Sham control	ICV (5 μl) PBS
2)	Disease control	ICV **Aβ**_**25–35**_ (5 μl) 500 nM
3)	Standard treatment	Memantine 5 mg/kg, p.o (28 days) + ICV **Aβ**_**25–35**_ (5 μl)
4)	Voglibose—low	1 mg/kg, p.o (28 days) + ICV **Aβ**_**25–35**_ (5 μl)
5)	Voglibose—high	10 mg/kg, p.o (28 days) + ICV **Aβ**_**25–35**_ (5 μl)

### Drugs and Treatment

Male Wistar rats (250–300 g) were divided into five groups with six animals each; sham control group (group I) with ICV administration of PBS; group II disease control group with ICV administration of Aβ_25–35_ [46], group III Aβ_25–35_ + memantine 5 mg/kg [47] (28 days), group IV Aβ_25–35_ + Voglibose 1 mg/kg (28 days), group V Aβ_25–35_ + Voglibose 10 mg/kg [28] (28 days). The entire grouping, dosing used in the study has been represented in Table 1. Similarly, the schedule and plan of the study has been represented in the Fig. 1.

### Behavioral study

#### Morris Water Maze

The behavioral test of Morris water maze (MWM) was performed to access spatial learning and memory in animals. Each animal was trained for three trials to reach the platform with aid of external cues for four consecutive days by gently placing the animals to the different quadrants with head facing to the wall. The animals were monitored for a period of 60 s. In case the animal was not able to find the platform, it was guided to the platform manually and allowed to stay for minimum 20 s to recognize the cues. Once the trial was done, it was dried gently using a clean towel and was gently placed back in the cage. The probe trial was performed on day 5 to evaluate whether the animals can reach the platform [48]. Using ANY MAZE software various parameters were evaluated such as D time, escape latency, path efficiency, and the number of times animals crossing the platform area were recorded.

#### Open Field Test

The open field test (OFT) is a method used in animal behavior research to measure post-surgical locomotor activity in rodents [49–51]. The open field arena is a square box made of opaque material, with high walls to prevent escape of 63*63*63 cm measurement with the floor marked with a grid to facilitate the analysis of the animal’s movement. The arena was cleaned thoroughly before each use to remove any residual scent or debris from previous animals. The animal was placed in the center of the arena and allowed to explore for 6 min of time. Several behavioral parameters such as, number of line crossings, grooming, and rearing were evaluated [52].

#### Novel Object Recognition Test

The novel object recognition test (NORT), a behavioral task commonly used in research studies to assess learning and declarative memory which measures the ability of rats to recognize a previously unseen object, and it involves three phases: habituation, training, and testing [53]. For the acclimation of all the rats to the test room, they were kept in the test room at least 30 min prior to the start of the experiment with the supply of free access to food and water, except during testing [54, 55]. The experiments were performed in the low light, sound-attenuated room setup between 8:00 and 20:00. Novel object recognition test was performed in an open field wooden box which is painted in black with a dimension of 63*63*63 cm. The box was cleaned and wiped with 70% isopropanol after the testing of each rat so that the smell of rodents does not have effect on the result. During the habituation phase, the rats were placed in an empty arena to explore and habituate to the environment for 10 min each. After 24 h in the training phase the rats were presented with two identical objects allowing them to explore them for 10 min. In this training period, it was noted that rats spent at least 30 s time exploring both the object by touching, sniffing, and rearing up to object. After 24 h, one of the familiar objects was replaced with a novel object. During the testing phase, the rats were again presented with the two objects for a period of 10 min, one familiar and one novel, and the time spent exploring each object was measured. The discrimination index (DI), a measure to distinguish two objects by rats, was calculated as difference between time spent by rat in novel object and old object divided by total time spent by rat in exploring novel and old object. DI = [novel (s) – familiar (s)]/[total novel (s) + familiar (s)] [54].

### Tissue Preparation for Acetylcholinesterase Activity and Western Blotting

The animals were euthanized, and the hippocampus and frontal cortex were isolated on an ice pack. Immediately, the tissues were homogenized. For acetylcholinesterase (AChE) activity, the tissues were homogenized with cold 1 × PBS pH 7.4 in ice-cold conditions and centrifuged in a cooling centrifuge at 10,000 RPM for 12 min to collect a clear supernatant used for the analysis. For western blotting, the tissues were homogenized using radioimmunoprecipitation assay buffer (RIPA buffer) containing protease and phosphatase inhibitor cocktail at 10,000 RPM for 10 min and centrifuged in a cooling centrifuge at 16,000 RPM for 20 min to obtain a clear supernatant. The estimation of total protein was done using a BCA protein assay kit (Cat. 23,225; Thermo fisher scientific).

### Estimation of AChE Activity

Modified Ellman’s method was used to estimate the AChE activity in the isolated brain samples. Acetylthiocholine is used as a substrate that is lysed by AChE to give thiocholine and acetate. The sulfahydryl group of thiocholine reacts with DTNB (5,5-Dithio bis (2-nitrobenzoic acid) to yield yellow color [56, 57]. The intensity of this yellow color is measured by ultraviolet (UV) spectrophotometer; the activity corresponds to the intensity of the color produced.

### Western Blot Analysis: Evaluation of WSP markers

The isolated rat brain was homogenized and processed for immunoblotting. Fifty micrograms of each protein was loaded in 8% polyacrylamide gel and separated at − 50 V for 2 h, followed by transfer into nitrocellulose membrane at − 90 V for 2 h [58–61]. The membrane was blocked for 2 h in 3% BSA in wash buffer (150 mM NaCl, 20 mM Tris base, 0.1% Tween 20; pH 7.6) followed by thrice washing in TBST and overnight incubation in 1:1000 dilution of primary antibodies [phospho-Tau(S396) polyclonal antibody (Cat: E-AB-68100; Elabscience); GSK3β polyclonal antibody (Cat: E-AB-67406; Elabscience); β-catenin polyclonal antibody (Cat: E-AB-15534; Elabscience); and β-actin polyclonal antibody (Cat: E-AB-30422; Elabscience) at 4 °C. The blots were then incubated with 1:10,000 dilution of horse radish peroxidase conjugated secondary antibody (Cat: E-AB-1003; Elabscience) for 2 h in room temperature, and finally, the peroxidase activity was visualized using ECL substrate kit. (Cat: XLS142.0250; Cyanagen, Italy).

### RTPCR Analysis: Evaluation of WSP Markers

The isolated rat brain were homogenized and the RNAiso plus reagent (Cat: 9108; Takara Bio Inc.) was used to isolate total RNA from the homogenate, following the instructions provided by the manufacturer [41]. Reverse transcription was done using 1 µg of total RNA with a primescript reverse transcription kit (Cat: RR037 A; Takara Bio Inc., India). The primers used for mRNA level estimation of rat brain were as follows: *Gsk3β* for qPCR (sense, forward 5'TCGCCACTCGAGTAGAAGAAA 3', antisense, 5'ACTTTGTGACTCAGGAGAACT3'); *Ctnnb1* for qPCR (sense, 5’-GTCTGAGGACAAGCCACAGGACTAC-3’, antisense, 5’- AATGTCCAGTCCGAGATCA GCA-3′); *Myc* for reverse transcription PCR (sense, 5′-CCAGCAGCGACTCTGAAGAAG-3′, antisense, 5′-GATGACCCTGACTCGGACCTC-3′); *Gapdh* (sense, 5′-GTGCCAGCCTCGTCTCATA-3′, antisense, 5′-GATGGTGQTGGGTTTCCCGT −3′). Expression levels of selected genes were measured by quantitative PCR (qPCR) analysis.

### Histopathology

The rats were euthanized and then their blood vessels were fixed by flushing normal saline through the heart, followed by a solution of 200 mL of 4% formaldehyde in phosphate buffer solution (0.1 M, pH 7.4) for 15 min. After that, the brains of the rats were taken out, separated, and fixed again in 4% formaldehyde for 24 h. To prepare paraffin embedded human brain sections, they were first treated by deparaffinizing with xylene and then rehydrated with a graded series of ethanol and distilled water. The hydrated slides were transferred to 50% alcohol for 30 s before being incubated in 50% ethanol with 0.01% sodium hydroxide in 50 ml for 5 min. Next, the slides were incubated in a 0.5% Congo red (CR) solution for 20 min. After CR incubation, the sections were incubated in 50% ethanol for 1 min, then in 70% ethanol for 1 min, and rinsed twice with fresh 95% ethanol for 2 min per rinse. They were then incubated with 100% ethanol twice for 2 min per rinse. Finally, the slides were rinsed in xylene twice for 3 min per rinse and covered with DPX (Cat: 06522; Sigma-Aldrich) mounting medium. The number of intact and damaged neurons in the hippocampal CA3 pyramidal layer were then counted using a light microscope (BX40, Olympus, New York, USA) connected to a camera (Olympus, DP12)[62].

### Statistical Analysis

The data is presented as mean ± standard error of the mean (SEM). To analyze the parameters of all the in vitro tests and estimates, a one-way analysis of variance (ANOVA) for independent measures was conducted, followed by post hoc Bonferroni’s multiple comparison test. Two-way ANOVA was used to analyze MWM data, followed by Bonferroni’s multiple comparison test. All other in vivo behavioral, biochemical estimations were analyzed using ANOVA followed by post hoc Bonferroni’s multiple comparison test. Statistical significance was set at *P* < 0.05.

## Results

### Evaluation of Neuroprotective Property of Selected Compounds in Differentiated SHSY5Y in Presence Glutamate

**Fig. 2:**
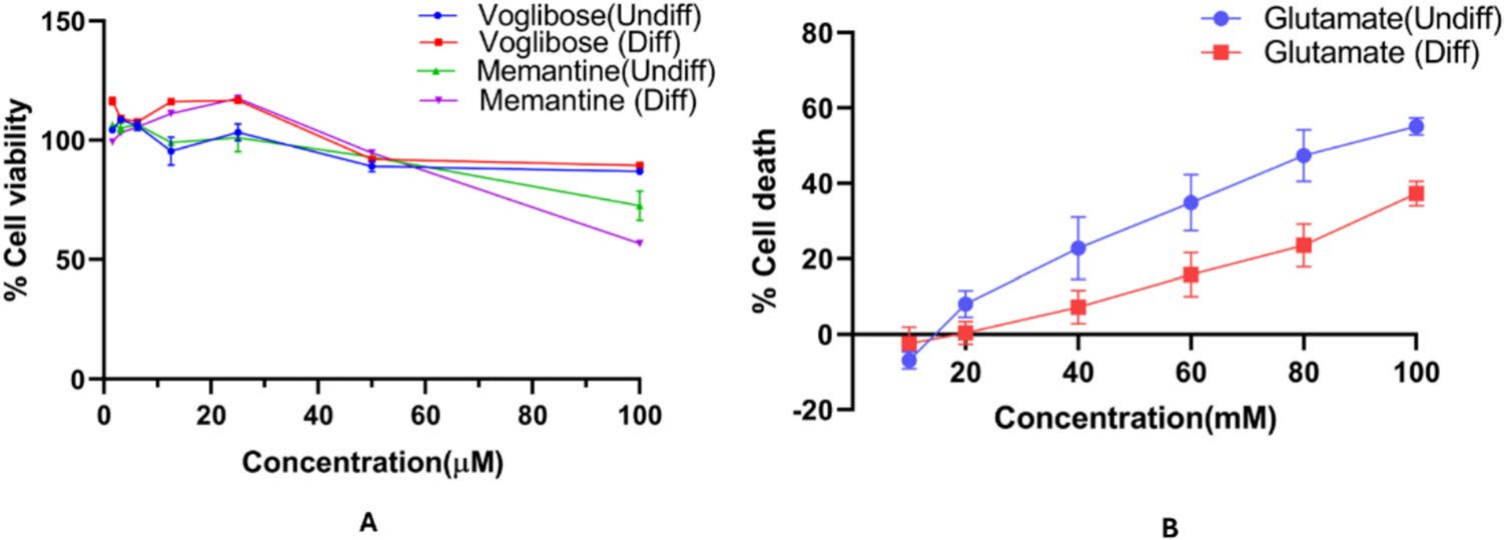
Neuroprotective activity (% cell viability) in glutamate (80 mM) induced neurotoxicity in and undifferentiated and differentiated SHSY5Y (mean ± SEM) with various dose and treatment strategies for 48 h followed by MTT assay

The RA differentiated SHSY5Y cells demonstrated greater protection from glutamate-induced toxicity when compared to the undifferentiated SHSY5Y cells. Specifically, RA-differentiated SHSY5Y cells exhibited 47.56% increase in cell viability at 100 mM concentration; 100% neuroprotection at 80 mM and 120% neuroprotection observed at 60 mM concentration of glutamate, compared to the undifferentiated SHSY5Y cells which can be observed in Fig. 2b. Memantine, a FDA-approved medication is used for AD that improves memory by its NMDA receptor antagonist property, acts by restoration of homeostasis in the glutamatergic system [63]. We have observed the maximum neuroprotection for Voglibose at 3.125 µM and memantine at 6.25 µM in undifferentiated SHSY5Y cells. Similarly, for differentiated SHSY5Y cells, the maximum neuroprotection was observed for Voglibose at 12.5 µM and memantine at 25 µM, as can be seen in Fig. 2a.

### Voglibose Treatment Exhibits Significant Reduction in the Binding of DKK1 to LRP6: DKK1 Binding Assay

**Fig. 3:**
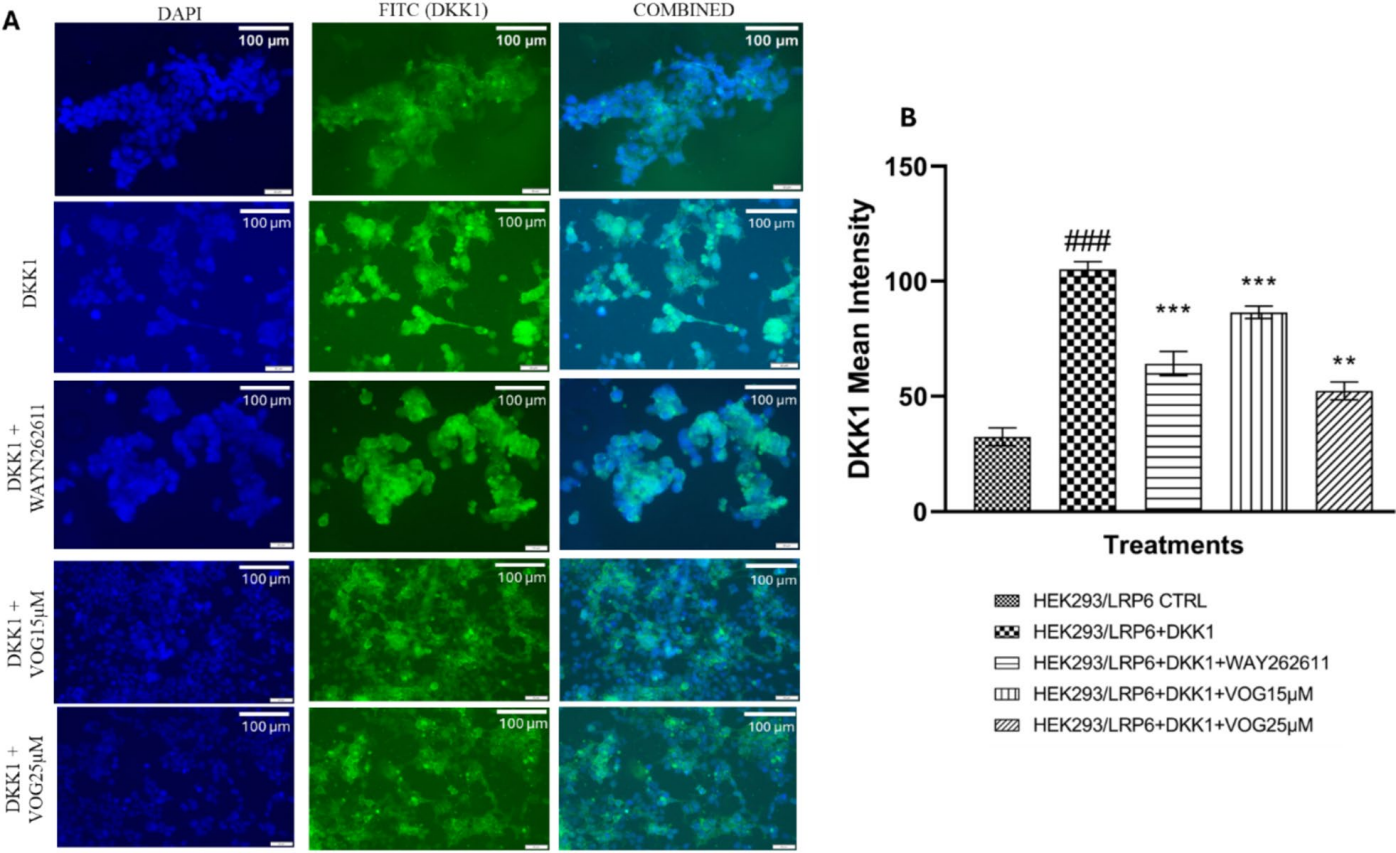
**A** Plot representing the immunostaining results for DKK1 binding in HEK293/LRP6 cells. (Scale bar 100 µm; 20 × magnification). **B** Quantification of the florescence for estimation of DKK1 binding. The data is presented as the mean ± SEM for three independent tests, each carried out in triplicate. Statistical analysis was performed using one-way ANOVA, followed by. Bonferroni’s multiple comparison test. ### represent *p* < 0.001 compared to HEK/LRP6 control group; **p < 0.01, and ***p < 0.001 compared to DKK1 treated HEK/LRP6 cells

LRP6 overexpressed cells showed minimum expression (not very significant) of DKK1 protein on its surface. The incubation of HEK293/LRP6 with DKK1 protein resulted in significant increase (105.20 ± 3.34 vs. 32.49 ± 3.84, *P* < 0.001) in the binding of DKK1 protein with the LRP6 coreceptor on the cell membrane surface as compared to control HEK293/LRP6 cells that can be observed in Fig. 3b as seen with increased green florescence in the cell membrane surface of cells. Cells which are treated with the known DKK1 inhibitor WAY262611 resulted in a significant decrease (64.17 ± 5.27 vs. 105.20 ± 3.34, F (4,35) = 53.56, *P* < 0.001) in the binding of DKK1 with LRP6 as compared to only DKK1 treated group of HEK293/LRP6 cells. WAY262611, chemically known as (1-(4-(naphthalen-2-yl)pyrimidin-2-yl)piperidin4-yl)methanamine, is a potent compound that prevents the binding of DKK1 to LRP6. In our current study, we have observed the reduction of fluorescence intensity in the DKK1 binding study for WAY262611 as well as both doses of Voglibose indicating these compounds have potential to inhibit binding of DKK1 to LRP6. Cells treated with Voglibose 25 µM showed significant (*P* < 0.0001) reduction in the binding of DKK1 (86.46 ± 2.66 vs. 105.20 ± 3.34, F (4,35) = 53.56, *P* < 0.01) as can be observed with the reduction in the intensity of florescence (green) in the Table 1. Similarly, the low dose treatment (15 µM) of Voglibose to DKK1 treated HEK293/LRP6 cells showed significant reduction (52.34 ± 3.86 vs. 105.20 ± 3.34, F (4,35) = 53.56, *P* < 0.001) in DKK1 binding with p value of < 0.01 which can be observed in the Fig. 3a.

### Significant Increased Expression of Canonical WSP Markers After Treatment with Voglibose: RTPCR Study

**Fig. 4:**
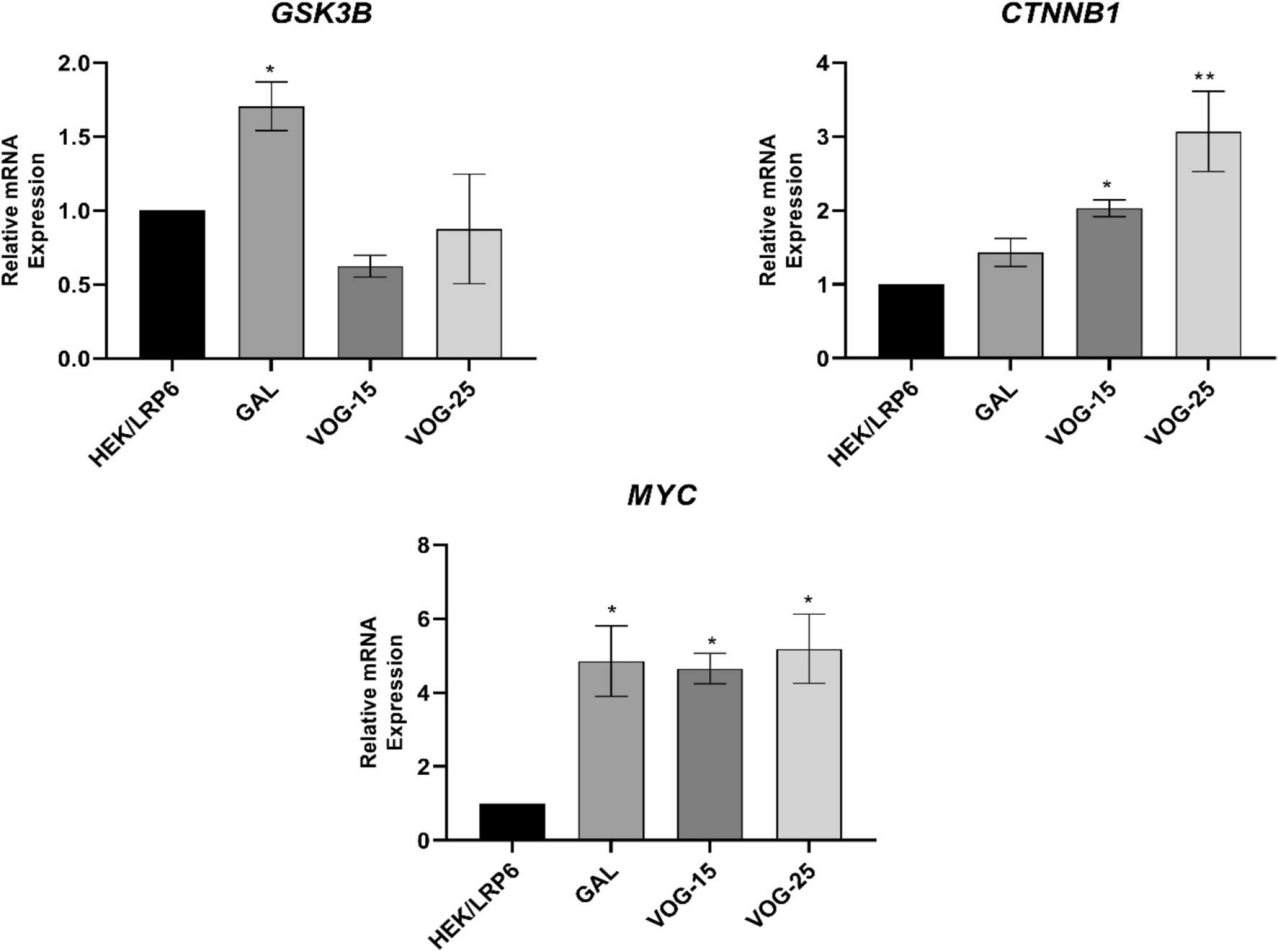
mRNA expression of *GSK3B*, *CTNNB1*, and *MYC*, after the 48 h treatment of different drugs in HEK293/LRP6 cells and evaluated using RTPCR. The data is presented as the mean ± SEM for three independent tests, each carried out in triplicate. Statistical analysis was performed using one-way ANOVA, followed by. Bonferroni’s multiple comparison test *p < 0.05, **p < 0.01 compared to normal HEK/LRP6 control cells

RTPCR study for the evaluation of gene level expression of canonical WSP markers was done in HEK293/LRP6 cells after treatment with Voglibose. The change in the expression of *GSK3B*, *CTNNB1*, and *MYC*, genes after the 48-h treatment of different drugs in HEK293/LRP6 cells has been represented in Fig. 4. Gallocyanine is a known DKK1 inhibitor which was used in our current study as standard for assessing DKK1 binding to assess WSP. The expression of *MYC* gene was found to be significant in the treatment of gallocyanine, (4.851 ± 0.957), *P* = 0.0236; Voglibose 15 µM (4.652 ± 0.4102), *P* < 0.05; and Voglibose 25 µM (5.186 ± 0.9385), *P* = 0.0149 as compared to control HEK293/LRP6 cells. *GSK3B* expression was reduced after the treatment with selected drugs, but significant change was not observed in any of the groups which is represented in Fig. 4. There was a significant increase in the expression of *GSK3B* in gallocyanin-treated HEK293 cells (1.707 ± 0.1641), *P* = 0.0243 when compared to the normal HEK293 cells. Similarly, decreased expression of *GSK3B* was observed after the treatment of Voglibose at concentration of 15 and 25 µM, but a significant chance was not seen. There was a significant increase in the *CTNNB1* level with the 15 µM Voglibose treatment (2.032 ± 0.1133), *P* = 0.0278 as well as 25 µM Voglibose treatment (3.070 ± 0.5415), *P* = 0.0017 when compared to HEK/LRP6 cells.

From these in vitro cell lines experiments that we have found the neuroprotective property of Voglibose in glutamate treated differentiated as well as non-differentiated SHSY5Y cells. Additionally, DKK1 binding assay using LRP6 overexpressed HEK293 cells has proven the potential of Voglibose to inhibit the binding of LRP6-DKK1 in comparison to standard WAYN262611. Similarly, gene expression study using RTPCR has proven the increased expression of genes related to canonical WSP which signifies the neuroprotective role of Voglibose through the modulation of canonical WSP. For further validation of these results, we have performed in vivo studies using Aβ_25–35_-induced AD rat model.

### Atomic Force Microscopy Reveals the Formation of Globular Oligomeric Species of Aβ_25–35_

**Fig. 5:**
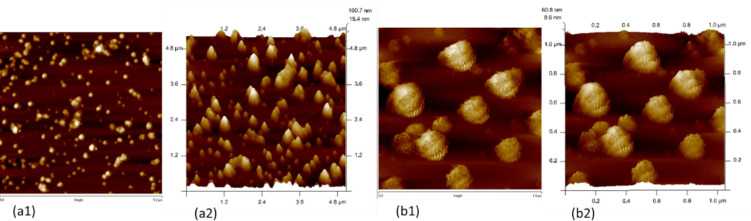
AFM analysis of the oligomer formation of Aβ_25–35_ a1. Aβ_25–35_ oligomeric formation as observed in 2D view at 5 µM resolution. a2. Aβ_25–35_ oligomeric formation as observed in 3D view at 5µM resolution. b1. Aβ_25–35_ oligomeric formation as observed in 2D view at 1 µM resolution. b2. Aβ_25–35_ oligomeric formation as observed in 3D view at 1µM resolution

Formation of globular-shaped oligomeric particles has been confirmed as seen in the AFM images in Fig. 5. The incubation of Aβ_25–35_ peptide with 1 × PBS for 72 h at 4 °C resulted in the formation of the oligomeric form of Aβ_25–35._ Analysis of the size distribution revealed that diameter of these oligomers were of size ranging from 115 to 435 nm with a mean diameter of 207 nm.

### MWM Study Reveals Improvement in the Spatial Memory in Aβ_25–35_-Induced Cognitive Decline in Voglibose Treated Rat Model

**Fig. 6:**
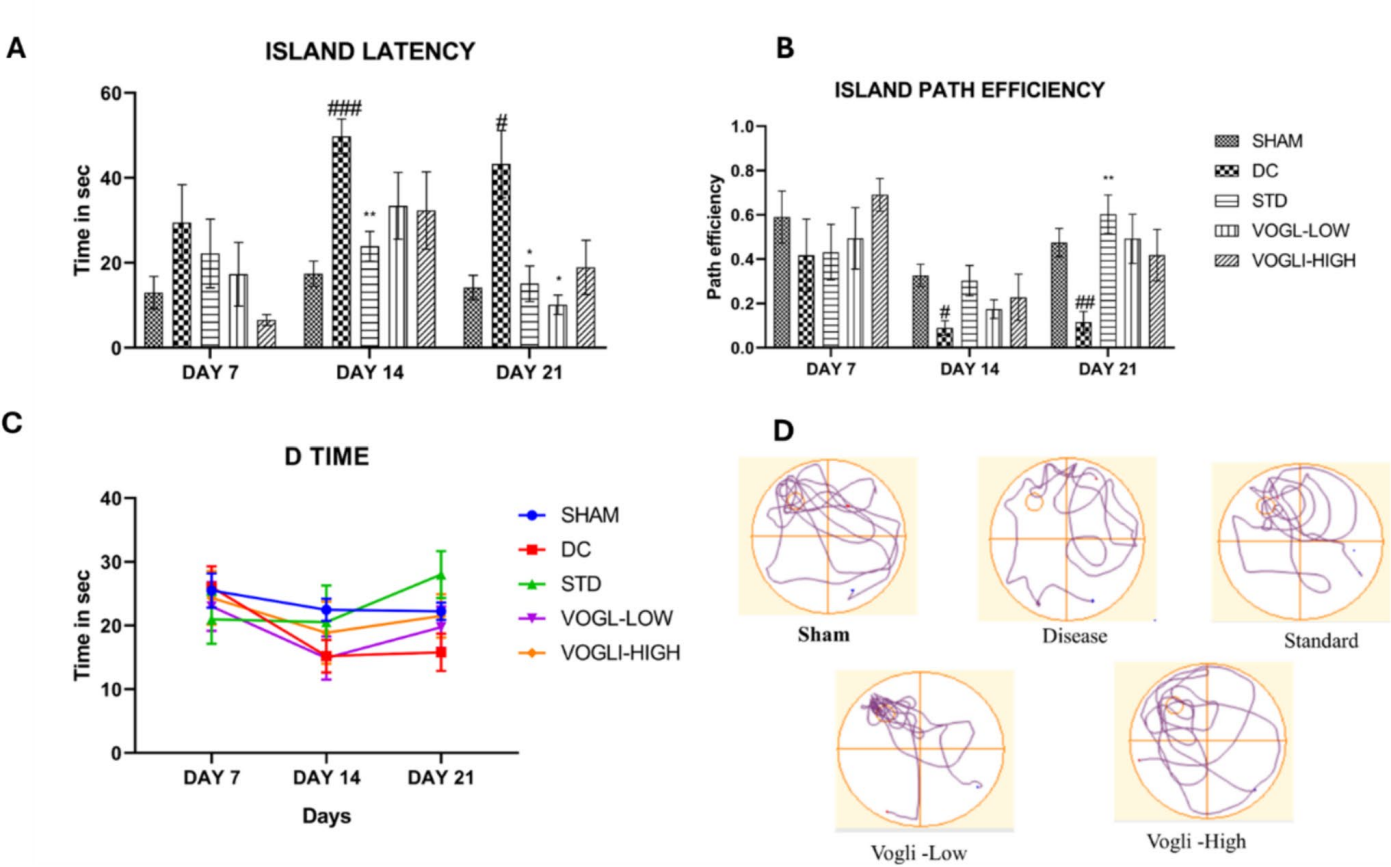
**a** Island escape latency, **b** island path efficiency, **c** time spent by animals in target “D” quadrant d. Track plot representation of animals during MWM as shown by animals with Voglibose treatment. Values are expressed as mean ± SEM (*n* = 6). Statistical analysis was done by using two-way ANOVA followed by Bonferroni multiple comparison test *p < 0.05, **p < 0.01, compared to disease control rats and #*p* < 0.05, ###*p* < 0.001 compared to sham control rats

Changes in the spatial memory in different groups of rats were carried out using MWM. The result from MWM has been represented in the figures below. Figure 6 represents island latency, time spent by animals in target quadrant, path efficiency shown by animals. The cognitive dysfunction was developed by injecting Aβ via the ICV route, which prominently increased the escape latency (49.77 ± 10.06 vs. 17.45 ± 7.258), *P* = 0.0004 and decreased path efficiency (0.089 ± 0.080 vs. 0.326 ± 0.1244), *P* = 0.0127 in comparison to the sham group on day 14. A similar trend was also observed on day 21 for island latency (43.25 ± 7.936 vs. 14.18 ± 2.873), *P* = 0.0378 and decreased path efficiency (0.1152 ± 0.04838 vs. 0.4745 ± 0.06436), *P* = 0.0048 in comparison to the sham group. Lower dose of Voglibose 1 mg/kg showed significant alteration in island latency (10.13 ± 2.273 vs. 43.25 ± 7.936, *F* (4,25) = 6.453, *P* = 0.0223) as compared with the disease group on day 21. Higher dose of Voglibose 10 mg/kg also showed decrease in island latency (18.395 ± 6.396, *P* = 0.1159) but was not significant (Fig. 6a). Voglibose 1 mg/kg showed increase (0.4917 ± 0.1104 vs. 0.1152 ± 0.04838, F (4,25) = 4.217, *P* = 0.0511) in the path efficiency and 10 mg/kg showed alteration (0.4180 ± 0.1165 vs. 0.1152 ± 0.04838, *P* = 0.1373) as compared to the disease group (Fig. 6b). Interestingly, the disease animals showed decrease in the D time (15.82 ± 2.921 vs. 22.25 ± 1.367) compared to the sham group, similarly low dose of Voglibose (19.73 ± 3.259 vs. 15.82 ± 2.921) and high dose (21.52 ± 3.390 vs. 15.82 ± 2.921) showed increased D-time, however, were insignificant compared to the disease group (Fig. 6c).

Figure 6d represents the track plot records of animals during MWM evaluation. It is evident that disease group animals showed reduced cognition shown by decreased entry to the island quadrant. As well as both the standard, memantine-treated animals and Voglibose-treated animals showed significant improvement in cognition and spatial memory.

### Impact of Experimental Procedure and Treatment on the Locomotion and Behavior in Rats: Behavioral Testing with OFT

**Fig. 7:**
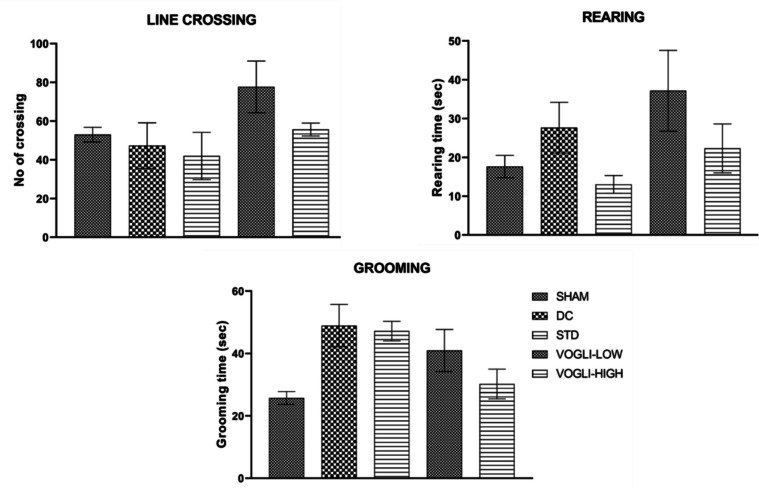
Number of line crossing, rearing and grooming activity of the rats treated with different doses of Voglibose in the open field test. Values are expressed as mean ± SEM (*n* = 6). Statistical analysis was done by using one-way ANOVA followed by Bonferroni multiple comparison test

Evaluation of normal behavior and locomotory changes in rats after surgical procedure was done using open field test (OFT) test. In this study, the disease group did not show much alteration in line crossing (47.33 ± 11.79 vs. 53.00 ± 3.786), rearing (27.67 ± 6.517 vs. 17.63 ± 2.867), and grooming (48.93 ± 6.779 vs. 25.75 ± 2.050) compared to the sham group the results were insignificant (Fig. 7). Low dose of Voglibose showed slight increase in line crossing (77.67 ± 13.37 vs. 47.33 ± 11.79), rearing (37.17 ± 10.42 vs. 27.67 ± 6.517), and decrease in grooming (40.95 ± 6.750 vs. 48.93 ± 6.779), in comparison to disease control however, it was not significant change. Similarly, high dose of Voglibose showed insignificant changes in line crossing (55.67 ± 3.333 vs. 47.33 ± 11.79), rearing (22.30 ± 6.300 vs. 27.67 ± 6.517), and grooming (30.20 ± 4.772 vs. 48.93 ± 6.779) as compared to disease control group. Rats treated with memantine showed insignificant changes in the line crossing (42.00 ± 12.17 vs. 47.33 ± 11.79), rearing (13.03 ± 2.281 vs. 27.67 ± 6.517), and grooming (47.20 ± 3.139 vs. 48.93 ± 6.779) behavior compared to the disease group. The nonsignificant changes observed in the number of line crossings in all the groups indicated that there were no changes in the locomotory behavior in rats after the ICV surgical procedure. Similarly, there were no significant changes in the rearing and grooming behavior of rats in different groups. This indicated the rats were not showing any anxiety and depression during the study duration.

### Exploratory and Discriminative Behavior in Rats: No Significant Treatment-Induced Changes in NORT

**Fig. 8:**
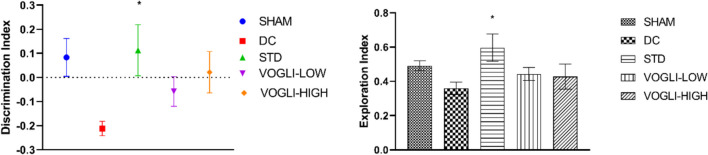
Effect of the different treatments on the novel object recognition test treated with the different doses of Voglibose. Values are expressed as mean ± SEM (*n* = 6). Statistical analysis was done by using one-way ANOVA followed by Bonferroni multiple comparison test. *p < 0.05 compared to disease control rats

NORT test was carried out to evaluate the changes in the episodic memory of rats after therapeutic treatments. During the training stage, it was discovered that all the rats explored identical things that were comparable to one another. When one object was later replaced with a new one, the rats receiving Aβ_25–35_ ICV injection showed a substantial decrease in sniffing and exploration time. In this study, the disease group showed a decrease in discriminative index (− 0.2116 ± 0.03004 vs. 0.08326 ± 0.07829) compared to the sham group. Rats in standard treatment arm (0.02196 ± 0.08580 vs. − 0.2116 ± 0.03004), *P* < 0.05 showed significant increase in the discriminative index compared to disease control group. Voglibose high dose (0.1130 ± 0.1058 vs. − 0.2116 ± 0.03004) and low dose (− 0.05799 ± 0.06192 vs. − 0.2116 ± 0.03004) treated group showed increased discriminative index but is not significant compared to the disease group (Fig. 8).

### Voglibose Treatment Exhibits Slight Reduction Without Significant Change in the AChE Activity

**Fig. 9:**
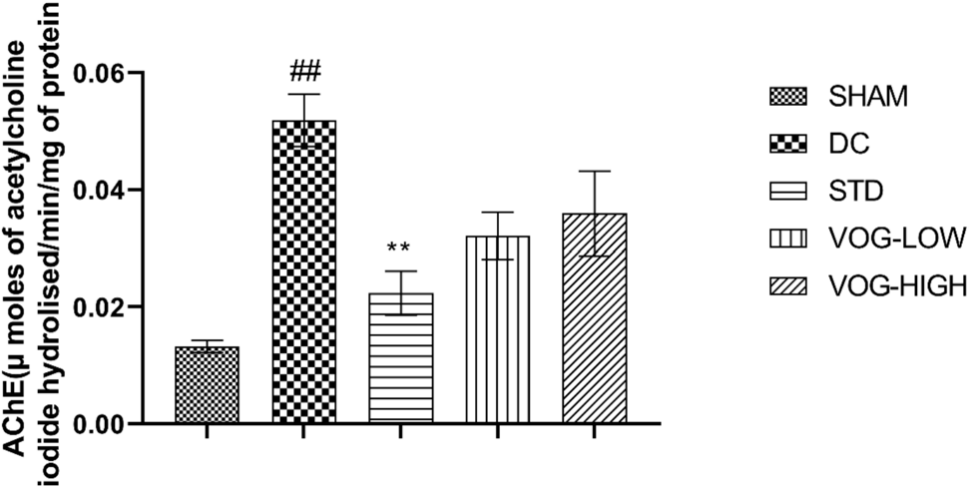
Acetylcholine level in the animal brain samples of the rats treated with different doses of Voglibose. Values are expressed as mean ± SEM (*n* = 6). Statistical analysis was done by using one-way ANOVA followed by Bonferroni multiple comparison test **p < 0.01 compared to disease control rats and ##*p* < 0.01 compared to sham control

AChE activity is one of the key hallmark of AD pathology. Since cholinergic deficits contribute to cognitive decline in AD, measuring AChE activity provides insights into disease progression. In this study, acetylcholinesterase levels are significantly increased in disease group (0.05184 ± 0.004472 vs. 0.01327 ± 0.001068, *P* = 0.0017) compared to the sham group. Insignificant changes were observed both in 1 mg of Voglibose (0.03211 ± 0.003993 vs. 0.05184 ± 0.004472) and 10 mg of Voglibose (0.03593 ± 0.007260 vs. 0.05184 ± 0.004472) compared to the disease group. Interestingly, standard dose significantly reduced the acetylcholinesterase levels (0.02236 ± 0.003786 vs. 0.05184 ± 0.004472, F (4,9) = 8.615, *P* = 0.0099) compared to the disease group (Fig. 9).

### Voglibose Treatment Mediated Reduction in Tau Phosphorylation and Activation of WSP: Western Blot Analysis

The level of β-catenin is indicator of the activated canonical WSP and thereby neuroprotective and cognitive effect. Here, significant reduction in the level of β-catenin (1.066 ± 0.006211 vs. 1.515 ± 0.08554, *P* < 0.05) was observed in Aβ-treated group compared to the sham group. Significant increase in the level of β-catenin has been observed in the rat brain treated with low dose of Voglibose (1.819 ± 0.1180 vs. 1.066 ± 0.006211, F (4,10) = 47.12, *P* < 0.05 compared to the disease group. However, rats treated with high dose of Voglibose showed decreased β-catenin level (0.5738 ± 0.05954 vs. 1.066 ± 0.006211, *P* < 0.05) compared to the disease group. Standard group also showed significantly increase (1.546 ± 0.02397 vs. 1.066 ± 0.006211, *P* < 0.05) in the β-catenin level compared to the disease group.

Similarly, significant increase in the level of p-tau (0.461 ± 0.04314 vs. 0.2311 ± 0.0234, *P* < 0.001) was observed in Aβ-treated group compared to the sham group. Significant decrease in the level of p-tau has been observed in the rat brain treated with low dose of Voglibose (0.2298 ± 0.007102 vs. 0.2311 ± 0.0234, F (4,10) = 63.43, *P* < 0.001, high dose of Voglibose (0.1978 ± 0.002631 vs. 0.2311 ± 0.0234, F (4,10) = 63.43, *P* < 0.001 compared to the disease group. Standard group also showed significant decrease (0.3314 ± 0.01733 vs. 0.2311 ± 0.0234, *P* < 0.05) in the β-catenin level compared to the disease group.

**Fig. 10:**
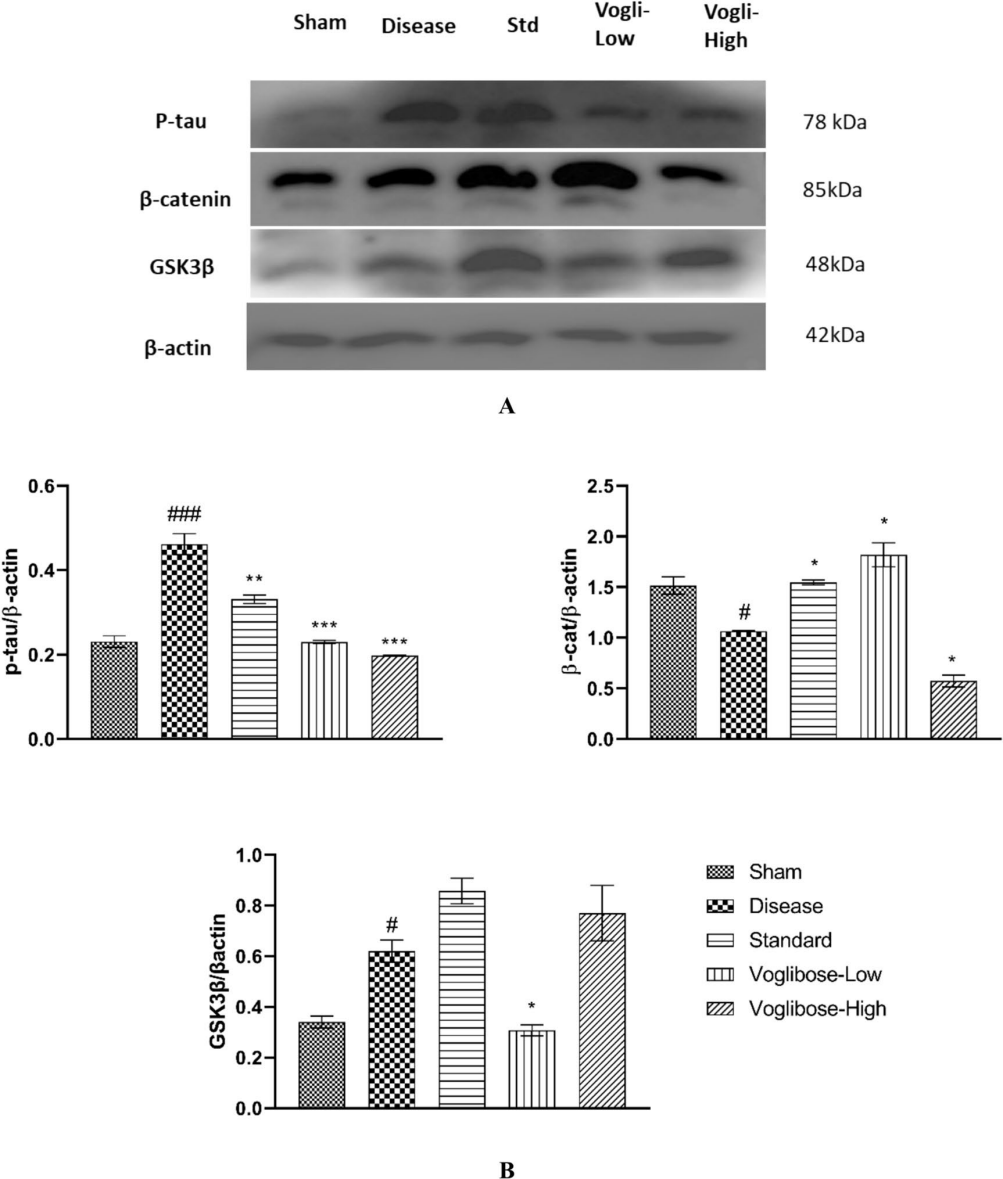
Voglibose regulated the expression of different protein levels involved in WSP in rodent model of AD. Representative image of blots showing expression of GSK3β, tau, and βcat markers, B. bar chart showing the relative expression of the makers. The quantification data were normalized using β-actin and the mean fold change in band intensity was compared to the disease and sham control group. Statistical analysis was done by using one-way ANOVA followed by Bonferroni multiple comparison test *p < 0.05, **p < 0.01, ***p < 0.001 compared to disease control rats; and #*p* < 0.05, ###*p* < 0.001 compared to sham control rats

GSK3β has been proven to play important role in the pathogenesis of AD. In this study, we found that Aβ-treated group significantly increased GSK3β (0.6202 ± 0.07571 vs. 0.340 ± 0.04085, *P* < 0.05) compared to the sham group. Interestingly, the level of GSK3β was also found to be increased in both standard treated and high dose of Voglibose treatment group as compared to disease control group. However, the level of GSK3β was significantly reduced in the rats treated with low dose of Voglibose (0.3076 ± 0.03674 vs. 0.6202 ± 0.07571, *P* < 0.05) as represented in Fig. 10.

### Voglibose Treatment Modulated the Activation of WSP in RTPCR Study

**Fig. 11:**
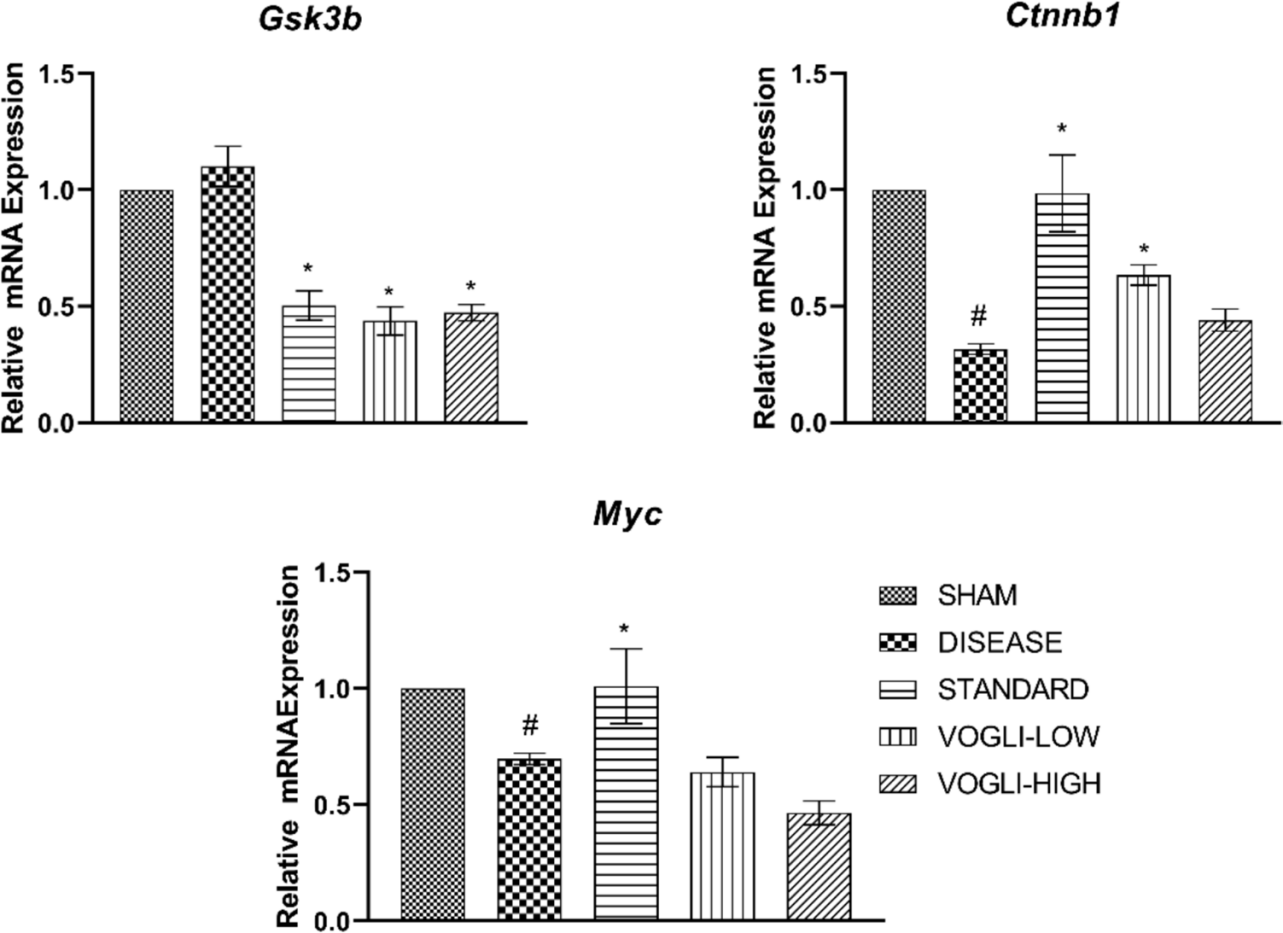
mRNA level expression of the genes involved in WSP (*Gsk3b*, *Ctnnb1 t*, and *Myc*) in the rats treated with different doses of Voglibose. Values are expressed as mean ± SEM (*n* = 6). Statistical analysis was done by using one-way ANOVA followed by Bonferroni multiple comparison test *p < 0.05compared to disease control rats; #*p* < 0.05 when compared to sham rats

Aβ-treated group showed an increase in the mRNA level of *Gsk3b* (1.102 ± 0.086) compared to the sham group. The significant reduction was observed in the mRNA level of *Gsk3b* in the rat brain samples treated with memantine (0.5032 ± 0.063 vs. 1.102 ± 0.086, *P* < 0.05), low dose of Voglibose (0.4377 ± 0.060 vs. 1.102 ± 0.086, *P* < 0.05) and high dose of Voglibose (0.4736 ± 0.035 vs. 1.102 ± 0.086, *P* < 0.05) when compared to the disease control group. Similarly, the brain samples of disease control rats treated with Aβ showed decrease in the level of *Ctnnb1* level compared to the sham group (*P* < 0.05). Rats treated with memantine (0.9859 ± 0.164 vs. 0.3152 ± 0.022, *P* < 0.05), low dose of Voglibose (0.6343 ± 0.044 vs. 0.3152 ± 0.022, *P* < 0.05) showed significant increase in the mRNA level of *Ctnnb1*, compared to disease control animals as represented in Fig. 11. However, rats treated with high dose of Voglibose showed an increase in mRNA level of *Ctnnb1*but not in the significant level. The level of Myc showed significant decrease in the disease control group as compared to sham control group. Both the group with Voglibose treatment did not show any significant increase in the level of Myc. However, the standard treated rats showed significant increase (1.010 ± 0.1611 vs. 0.6974 ± 0.023, *P* < 0.05 in the *Myc* level when compared to disease control group.

### Voglibose Reduced the Expression of Amyloid Deposits in the Cortex and Hippocampal Regions of AD Rats: Histopathological Analysis

**Fig. 12:**
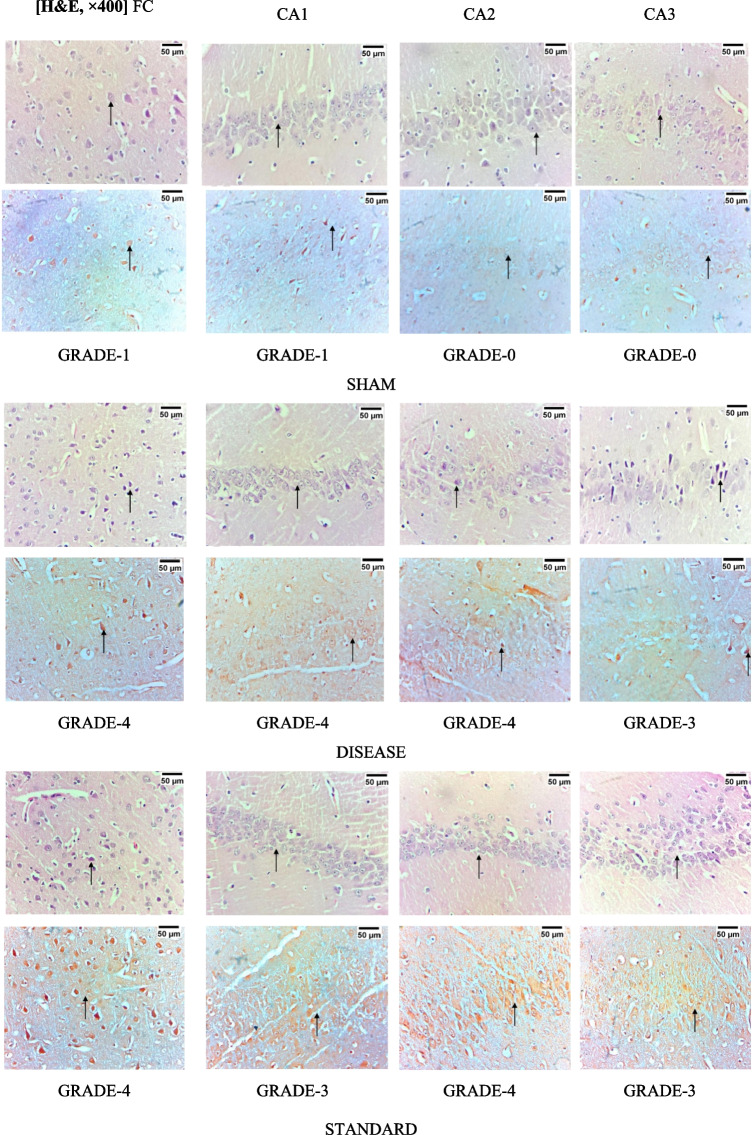

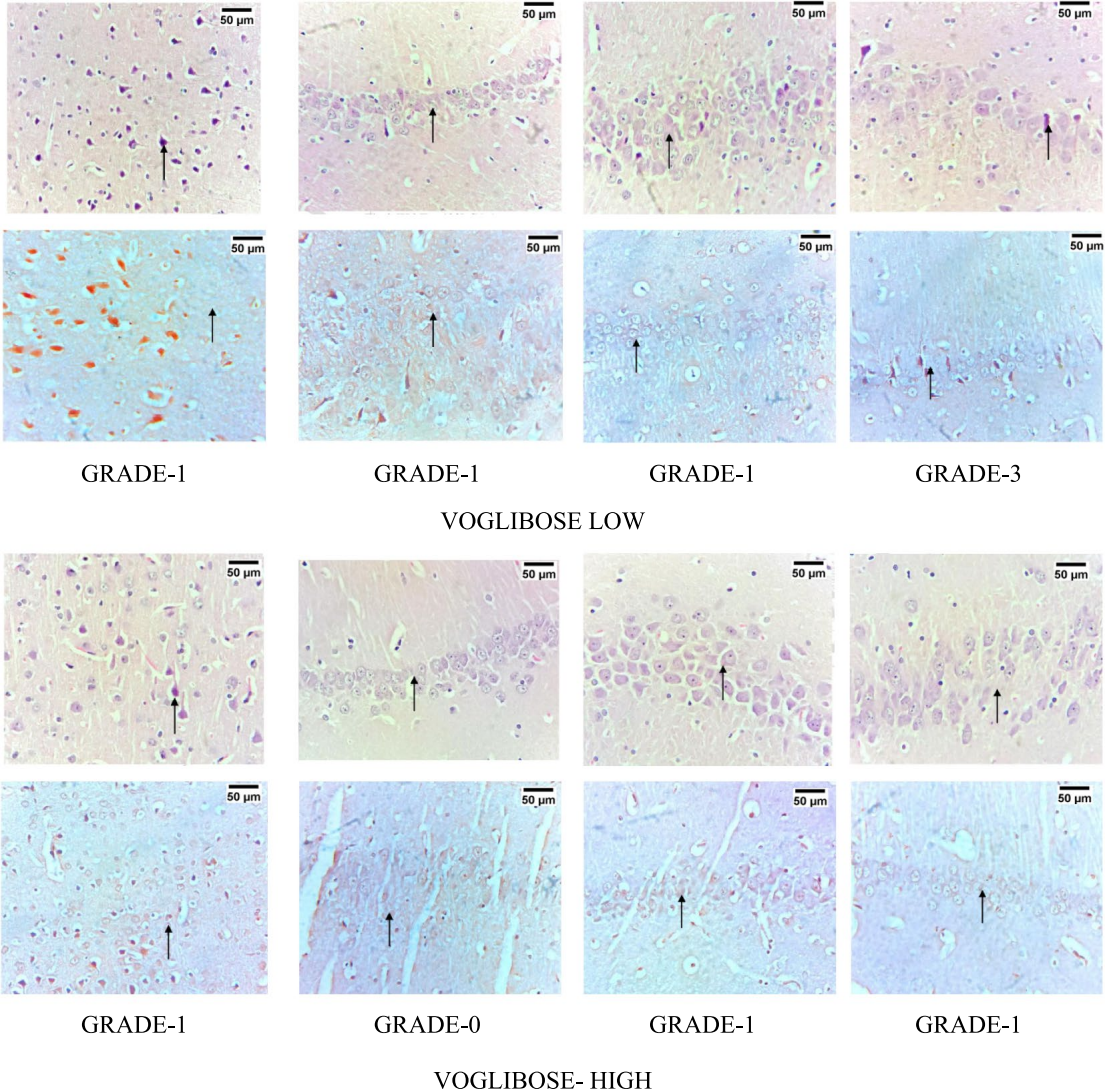
Effect of Voglibose on amyloid deposition in rat brain regions. Photomicrographs of different brain regions were taken at 40 × magnification; Scale: 50 µm. The grading system for amyloid presence is as follows: grade 0 indicates no amyloid deposition; grade 1 represents minimal amyloid (< 10% deposition); grade 2 indicates focal deposition (11–25%); grade 3 represents moderate deposition (26–50%); grade 4 indicates diffuse deposition (51–75%); and grade 5 represents extensive deposition (> 75%)

The histopathological staining of the different regions of the brain including frontal cortex; CA1, CA2, and CA3 region of the hippocampus has been represented in Fig. 12. Different grading has been provided to the presence of amyloid as grade 0 as no amyloid; 1 representing minimal amyloid with < 10% amyloid deposit; 2 indicating focal deposition with 11–25% of amyloid deposition; 3 indicating moderate deposition with 26–50% of amyloid deposition; 4 indicating diffused deposition of amyloid with 51–75% deposition; and 5 representing extensive deposition of amyloid with > 75% amyloid. Disease control rats treated with amyloid 25–35 exhibited extensive deposition of amyloid in both hippocampal and frontal cortical region. Both the high dose as well as low dose treatment with Voglibose exhibited decreased deposition of amyloid beta.

### Voglibose Showed No Change in the Random Blood Sugar Level

**Fig. 13:**
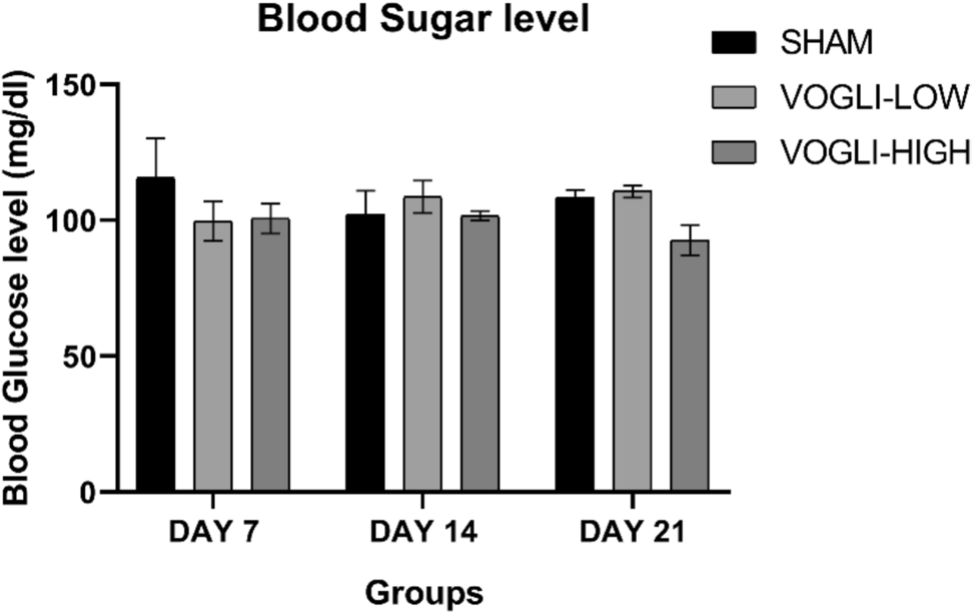
Blood sugar level of the rats treated with different doses of Voglibose. Values are expressed as mean ± SEM (*n* = 6). Statistical analysis was done by using one-way ANOVA followed by Bonferroni multiple comparison test

For the evaluation of changes in the blood sugar level in rats after treatment with antidiabetic drug, we have measured blood sugar level at different intervals in our study. The blood sugar level in all the rats treated with Voglibose did not show any significant change on day 7, 4, and 21 shown in Fig. 13. There was slight reduction in the blood sugar level in rats treated with the high dose of Voglibose on day 21; however, it was not significant level as compared to the sham group. The dose used in the current study did not cause significant hypoglycemic effect throughout the study duration.

## Discussion

The current study’s findings showed that a low dose of Voglibose significantly affected the participants'spatial cognitive performance on the Morris water maze task. Voglibose therapy significantly reduced mean escape latency compared to disease control rats thereby improving the retention memory of the animals. Voglibose treated rats have spent more time in the target quadrant and finding the hidden platform. The results of this investigation aligns with earlier research demonstrating that Voglibose therapy enhances spatial learning and memory performance in MWM [28, 64]. It also reduced the expression of GSK-3β and increased the levels of β-catenin indicating activation of WSP. β-catenin, a key mediator of the canonical WSP pathway, is crucial for neurogenesis, long-term potentiation (LTP), and hippocampal function, all of which are essential for the consolidation of spatial memory in MWM. β-catenin regulates synaptic strength by reinforcing dendritic spines and promoting synapse formation, thereby improving learning and spatial memory [65]. In AD, several research indicates that Aβ stimulates neuronal death by activation of GSK-3β, leading to breakdown of β-catenin and inactivation of WSP [44]. Our findings suggest that Voglibose modulates the canonical WSP pathway, which is critical for synaptic plasticity and memory formation. This mechanism may also contribute to a reduced discrimination index in NORT by stabilizing β-catenin, thereby promoting neuronal survival and synaptic remodeling. These results highlight the potential neuroprotective effects of Voglibose through modulation of WSP, extending beyond its primary role in glycemic control [66].

Cells after differentiation with retinoic acid were resistant and protected from toxicity of glutamate. It has been proven that membrane lipid cholesterol density gets reduced after the cell differentiation which might be due to the elongation of neurite supporting the fact that differentiated cells show resistance to glutamate even in our study [67]. In LRP6 overexpressed HEK293 cells, we have proven that Voglibose-reduced DKK1 binding using immunofluorescence method. DKK1 inhibition results in the increased level of cytoplasmic β-catenin level and thereby activation of canonical WSP [68, 69]. In the present study, we found increased mRNA level of *MYC* which is a transcriptional gene involved in the cell growth, cell cycle, self-renewal, metabolism, and differentiation [70, 71] indicting the activation of WSP by both does of Voglibose. Gallocyanine, a DKK1 inhibitor was used to assess WSP in neuroprotection. By inhibiting DKK1, it may enhance synaptic plasticity and cognitive resilience, highlighting the therapeutic potential of the DKK1-Wnt axis in neurodegenerative disorders. [39] The comparable result was observed in the gene level estimation between in vitro and in vivo experiments; a significant decrease was observed in the level of *GSK3B* in both experiments with both higher and lower dose of Voglibose. For *CtnnB1* and *Myc* level, the higher dose of Voglibose did not show significant increase in rodent model whereas in cell line study there was significant increase in expression. However, the lower dose of Voglibose showed significant increase in *Ctnnb1*expression in the rodent model.

The primary types of Aβ present in the human brain are Aβ_1–42_. However, the Aβ_25–35_ fragment, which is a natural occurrence in older individuals, is more toxic and is now recognized to have a significant role in AD due to its unique ability to aggregate. [72] Aβ_25–35_ is thought to be the functional area of Aβ that is accountable for its neurotoxic characteristics. It is the biologically active segment of Aβ. When given to mice, Aβ_25–35_ has been demonstrated to cause memory loss, impair spatial working memory, and break down passive avoidance reactions [73]. In the current study, we have used oligomeric Aβ_25–35_ by intracerebroventricular (ICV) injection to induce AD in rodent model. Induction of memory impairment started after 14 days of ICV as shown by island latency in Moris water maze test (MWM) test.

The WSP plays a crucial role in the nervous system, and its dysregulation has been implicated in the pathogenesis of neurodegenerative diseases [74]. Many studies have focused on insulin, its signaling, and resistance in both peripheral and central tissues as the primary pathological mechanism linking AD and diabetes [21]. When insulin binds to its receptor, it activates insulin signaling, which subsequently activates the PI3 K and Akt signaling pathway, leading to the inhibition of GSK-3β activity. In AD, the level of GSK-3β is elevated, and it is involved in the degradation of β-catenin and inactivates the WSP [22, 23]. GSK-3β, a key component of the Wnt pathway, contributes to the accumulation of tau inclusions [75]. In the early stages of AD, phosphorylated tau aggregates within neurons, forming pre-tangles that precede neurofibrillary pathology [76]. Our results align with previous literature, showing that Aβ accumulation leads to increased tau phosphorylation. Notably, Voglibose reduces tau phosphorylation and mitigates its pathogenic effects. AChE activity was assessed to evaluate cholinergic dysfunction, a key hallmark of AD pathology [77, 78]. Since cholinergic deficits contribute to cognitive decline in AD, measuring AChE activity provides insights into disease progression. In the current study, AChE levels are significantly increased in the disease group compared to the sham group and standard drug significantly reduced the AChE levels. However, the AChE levels in the low and high dose treatment groups of Voglibose were not significantly different from the disease group (Fig. 9). This could be due to the fact that Voglibose primarily acts on glucose metabolism and gut brain interactions. Its impact on AChE might be secondary and require prolonged treatment.

The amyloid hypothesis, neuroinflammation, oxidative stress, insulin resistance, and mitochondrial abnormality have all been associated with AD [7]. Additionally, the loss of the standard WSP has been linked to AD’s development. Various studies have focused on activating WSP using different strategy as a treatment for AD [30, 79–81]. Cisternas et al.’s in vivo experiments have demonstrated that activating the WSP in hippocampal neurons promotes the utilization and uptake of glucose [82]. The β-catenin dependent WSP inhibits adipogenesis, stimulates myogenesis, and increases muscle glucose uptake. Activating the canonical WSP has been found to enhance glucose metabolism and memory in the APPswe/PS1E0 transgenic mouse model by increasing AMPK mediated activity [83]. Voglibose enhances the WSP by preventing interaction of DKK1 with LRP6 thereby reducing GSK3β and reducing tau hyperphosphorylation. APP-induced phosphorylation of β-catenin reduces total β-catenin levels, indicating that APP expression facilitates β-catenin degradation. In our results, Voglibose reverses the effect through WSP mechanisms. Overall, our findings underscore the potential of Voglibose in mitigating cognitive deficits, possibly through modulation of the WSP. These results suggest that Voglibose may offer neuroprotective advantages in addition to its antidiabetic actions, making it a promising candidate for further investigation in the context of neurodegenerative diseases such as AD.

The accumulation of insoluble amyloid plaques in nerve tissue is believed to be the cause of neurodegenerative diseases like AD and Parkinson’s. One of the gold standard staining methods to detect these protein aggregates is to use CR [84, 85]. CR is a direct diazo dye primarily used to color paper products [86]. The molecular docking simulation results indicate that CR binds to sites parallel to the fibril axis and antiparallel to the β-sheets on amyloid fibrils and protofibrils [87]. We have used CR staining techniques to identify amyloid deposits in the brain region, where both the treatment doses of Voglibose have showed reduced deposition of amyloid. CR stanning has been identified to show non-specific binding to other aggregates, elastotic dermis, hyaline deposits in colloid milium and in lipid proteinosis [84]. However, to further strengthen the findings, we acknowledge that additional amyloid-specific staining techniques, such as thioflavin S or anti-Aβ immunohistochemistry, could provide complementary confirmation.

While this study provides valuable insights into the role of DKK1 inhibition and WSP modulation, certain limitations should be acknowledged. Firstly, although qPCR analysis of *GSK3B* and MYC offers a transcriptional perspective, protein level validation through western blotting or immunostaining was not performed, which could have strengthened the mechanistic findings. Additionally, while CR staining was used to assess amyloid aggregation, further quantitative analyses or complementary staining methods could enhance the robustness of these findings. Moreover, brain-derived neurotrophic factor (BDNF) treatment which could have provided insights into neurotrophic support and synaptic plasticity was not performed. The study primarily focuses on HEK cells, and further validation in neuronal or disease relevant models would improve translational significance. Another limitation is the lack of functional assays to directly assess synaptic plasticity or neuronal survival, which would provide a more comprehensive understanding of the neuroprotective effects. Another limitation is the relatively small sample size [*n* = 6, per group], use of male rats, in vivo experiment with two doses which can increase the potential for variability in the behavioral assay and may affect the statistical robustness. In current study, we have not accessed the possible side effect of Voglibose in the rodent model. Further studies incorporating protein-level validation, BDNF treatment, additional cellular models, more comprehensive models, safety profile, and functional analyses will help to further elucidate the therapeutic potential of DKK1 inhibition in neurodegeneration. Additionally, currently, our study focuses on WSP pathway; future studies could explore other possible mechanisms of activity for memory enhancing properties of Voglibose in greater depths.

## Conclusion

The exact pathophysiology involved in AD is still a topic of debate and a challenge for many researchers. Late diagnosis as well as lack of therapeutic treatments adds another hurdle for the patients suffering from AD. In current scenario of lack of disease modifying therapeutics for AD and failures of several new molecules in early stages of clinical trial, repurposing of known FDA approved drugs holds immense potential. Linking diabetes and AD has been seen in many older patients. Currently, by utilizing in vitro and in vivo tools, we have demonstrated that Voglibose showed potential cognitive and neuroprotective property, mediated through WSP pathway. Further validation of the study is warranted using more detailed procedures as well as in clinical studies.

## Supplementary Information

Below is the link to the electronic supplementary material.

Supplementary file1 (DOCX 6806 KB)

## Data Availability

No datasets were generated or analysed during the current study.
