# Supplementary material for: Voglibose Attenuates Amyloid Beta–Induced Memory Deficits in a Rodent Model: A Potential Alzheimer’s Therapy via Wnt Signaling Modulation
Source: Mol Neurobiol. 2025 May 17;62(9):12184–204. doi: 10.1007/s12035-025-05047-5 (PMC12367840; doi:10.1007/s12035-025-05047-5)
Supplement: Supplementary file 1 — Supplementary file1 (DOCX 6806 KB) [file 12035_2025_5047_MOESM1_ESM.docx]

### **Suppliment**

### **S1. SH-SY5Y cell culture and neuronal differentiation using Retinoic acid:**

SHSY5Y cells get typical differentiated with elongated cells and extended neurite length after two days of treatment with retinoic acid which can be observed in the S1 below.

| 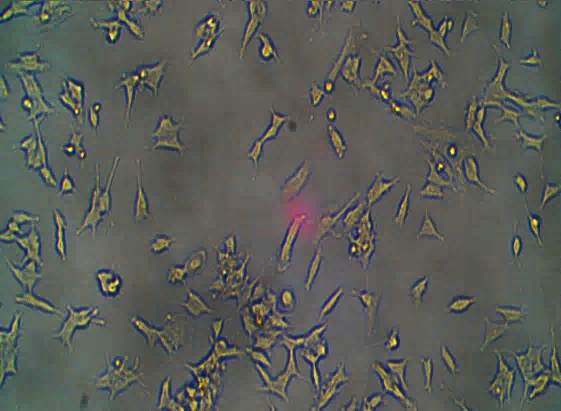  Day 1 | |
| --- | --- |
| 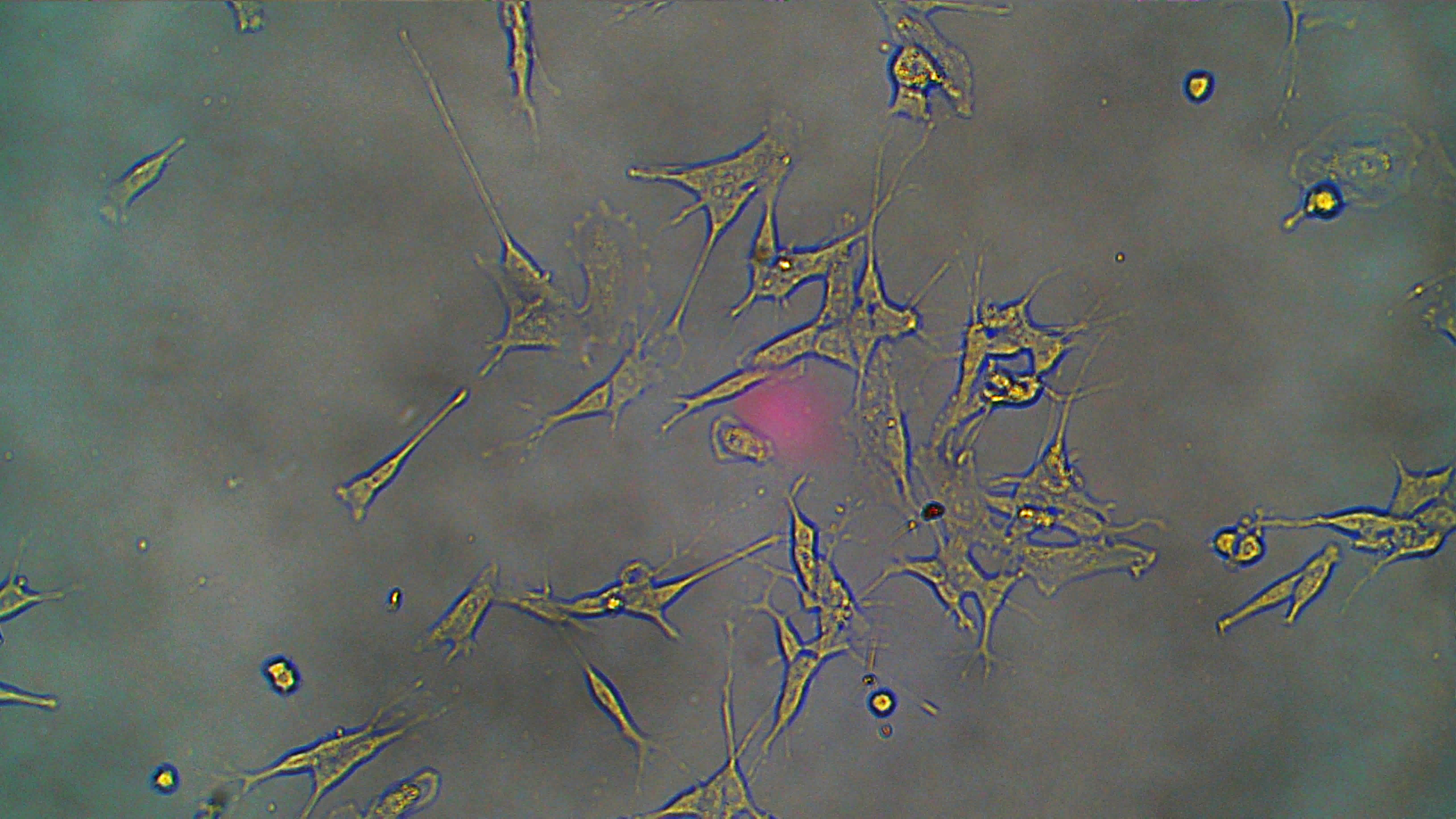  Day 2 | 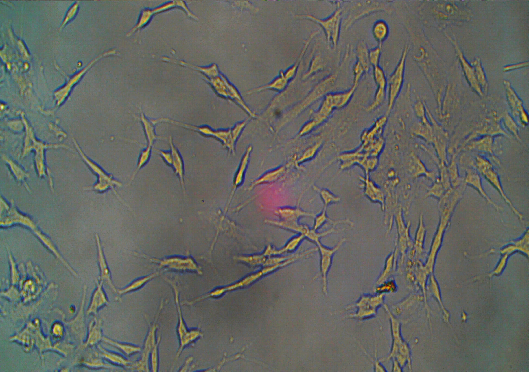  Day 3 |
| 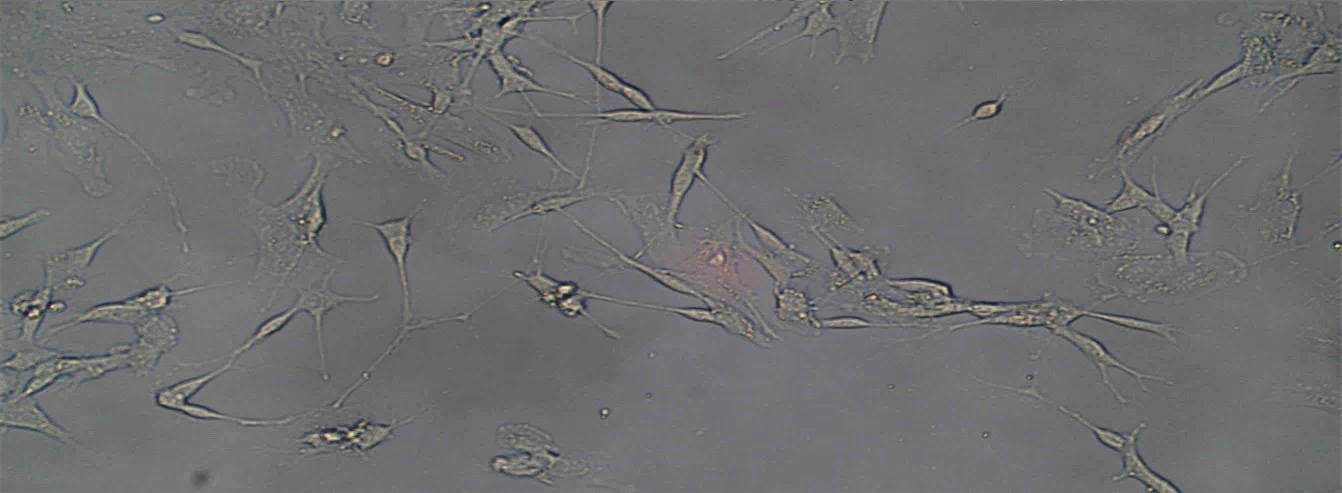  Day 4 | 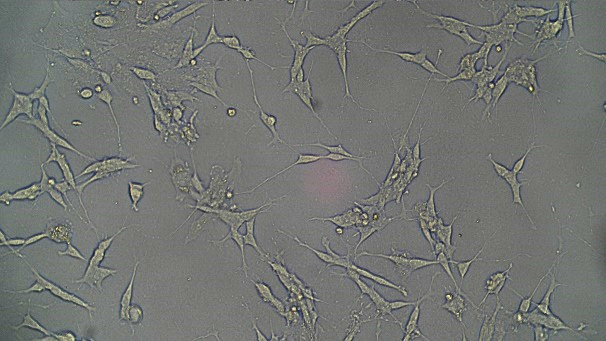  Day 5 |

S1 Representative image showing the extra growth of neurites in SH-SY5Y cells on treatment with 10µM retinoic acid for 5 consecutive days.

Supplementary table 1. Representative image showing the blots for the expression of different protein levels involved in WSP in rodent model of AD.

| p-tau  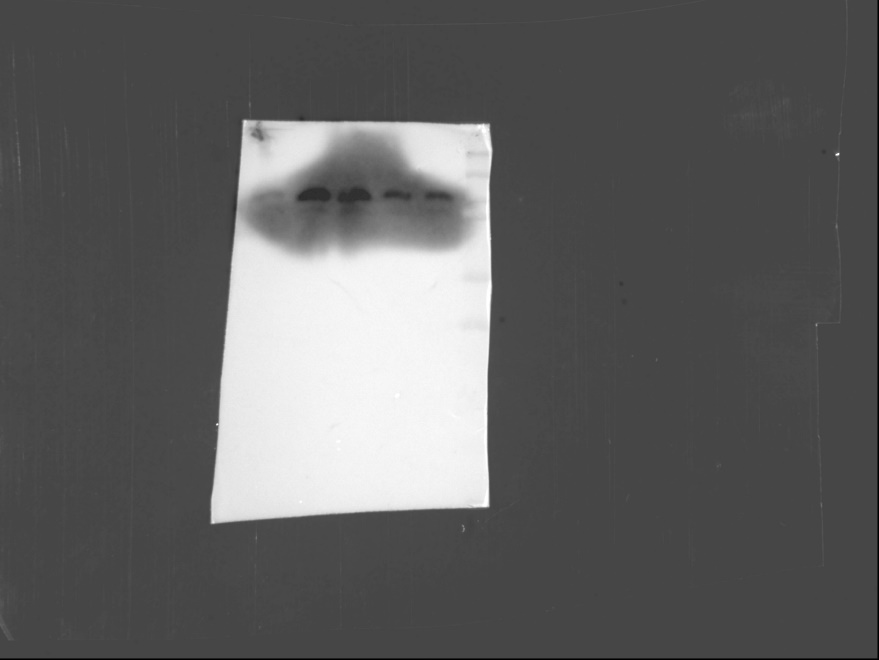 | β-actin  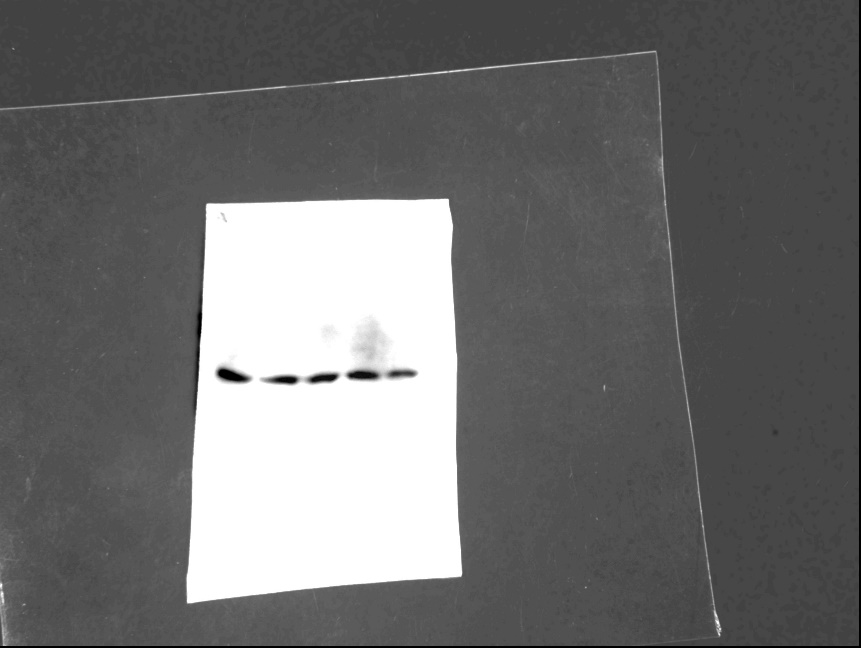 |
| --- | --- |
| GSK-3β  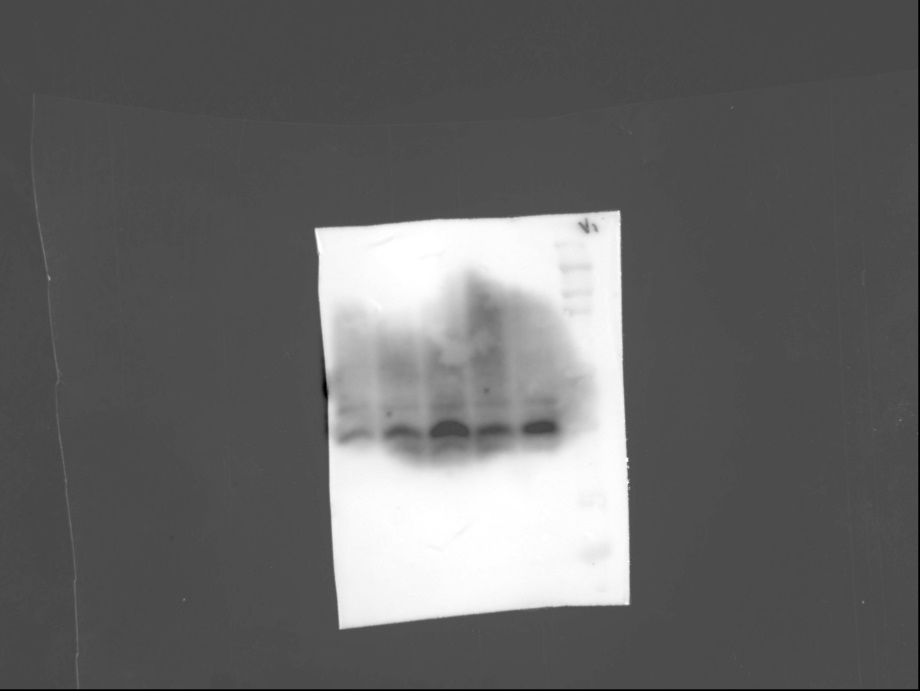 | β-actin  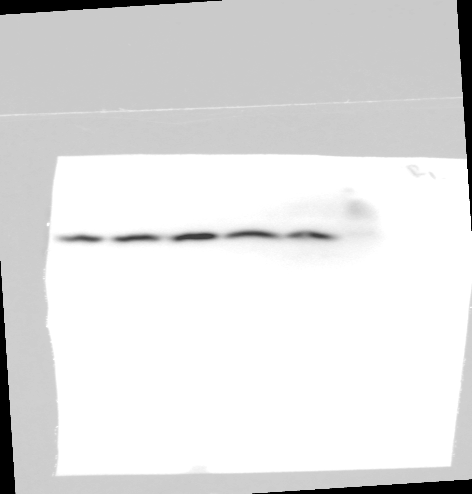 |
| β -cat  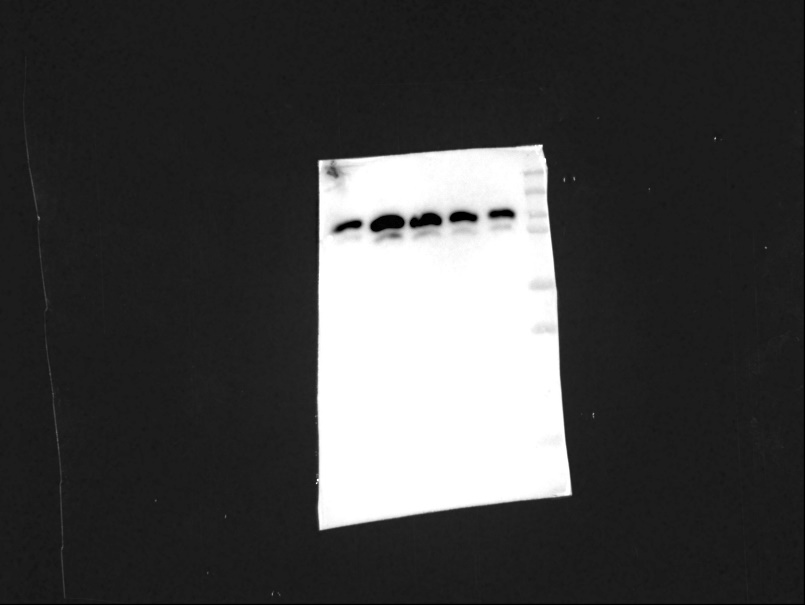 | β-actin  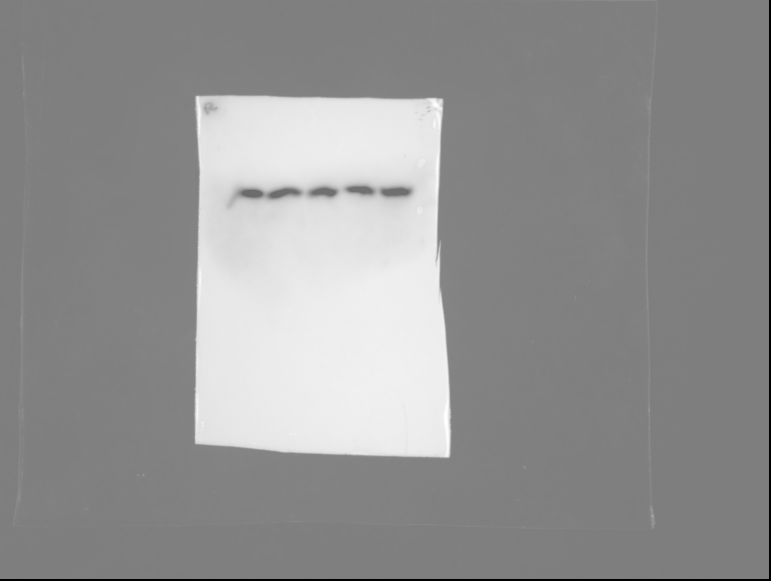 |
